# An Update on the Progress of Isolation, Culture, Storage, and Clinical Application of Human Bone Marrow Mesenchymal Stem/Stromal Cells

**DOI:** 10.3390/ijms21030708

**Published:** 2020-01-21

**Authors:** Dinh-Toi Chu, Thuy Nguyen Thi Phuong, Nguyen Le Bao Tien, Dang Khoa Tran, Vo Van Thanh, Thuy Luu Quang, Dang Tien Truong, Van Huy Pham, Vo Truong Nhu Ngoc, Thien Chu-Dinh, Kushi Kushekhar

**Affiliations:** 1 Faculty of Biology, Hanoi National University of Education, Hanoi 100000, Vietnam; 2 School of Odonto Stomatology, Hanoi Medical University, Hanoi 100000, Vietnam; votruongnhungoc@gmail.com; 3 Department of Animal Science, College of Agriculture and Life Science, Chonnam National University, Gwangju 61186, Korea; 4 Institute of Orthopaedics and Trauma Surgery, Viet Duc Hospital, Hanoi 100000, Vietnam; bstiencsvd@gmail.com (N.L.B.T.); thanhvo@hmu.edu.vn (V.V.T.); 5 Department of Anatomy, University of Medicine Pham Ngoc Thach, Ho Chi Minh City 700000, Vietnam; khoatrandr@gmail.com; 6 Department of Surgery, Hanoi Medical University, Hanoi 100000, Vietnam; 7 Center for Anesthesia and Surgical Intensive Care, Viet Duc Hospital, Hanoi 100000, Vietnam; drluuquangthuy@gmail.com; 8 Vietnam Military Medical University, Hanoi 100000, Vietnam; truongdtvmmu@gmail.com; 9 AI Lab, Faculty of Information Technology, Ton Duc Thang University, Ho Chi Minh City 700000, Vietnam; 10 Institute for Research and Development, Duy Tan University, Danang 550000, Vietnam; 11 Institute of Cancer Research, Oslo University Hospital, 0310 Oslo, Norway; kushi.kushekhar@medisin.uio.no

**Keywords:** bone marrow mesenchymal stem cells, stem cell therapy, isolation, culture, storage, clinical application

## Abstract

Bone marrow mesenchymal stem/stromal cells (BMSCs), which are known as multipotent cells, are widely used in the treatment of various diseases via their self-renewable, differentiation, and immunomodulatory properties. In-vitro and in-vivo studies have supported the understanding mechanisms, safety, and efficacy of BMSCs therapy in clinical applications. The number of clinical trials in phase I/II is accelerating; however, they are limited in the size of subjects, regulations, and standards for the preparation and transportation and administration of BMSCs, leading to inconsistency in the input and outcome of the therapy. Based on the International Society for Cellular Therapy guidelines, the characterization, isolation, cultivation, differentiation, and applications can be optimized and standardized, which are compliant with good manufacturing practice requirements to produce clinical-grade preparation of BMSCs. This review highlights and updates on the progress of production, as well as provides further challenges in the studies of BMSCs, for the approval of BMSCs widely in clinical application.

## 1. Introduction

Stem cell therapy is considered as a powerful tool for various clinical applications. Human stem cells can be obtained from different tissues in adults during their lifetime, including bone-marrow mesenchymal stem cells (BMSCs) [1], umbilical cord stem cells (UCSCs), adipose-derived stem cells (ADSCs), skeletal stem cells, and other types, such as dental pulp, skin, and placenta [2,3,4,5,6,7]. Some studies have defined the stem cells from bone marrow as standard mesenchymal stem cells (MSCs). The International Society for Cellular Therapy (ISCT) defines the minimal criteria of mesenchymal stem cells [8]. MSCs have been demonstrated to differentiate into different cell lineages which regulate the immune system and induce anti-inflammation [9]. During treatment, the infused MSCs can bring effects on injured or inflammation sites. MSCs expressed far-distance effects by secreted bioactive factors. Stem cell therapy works as a medicine. MSCs are also accepted the name of mesenchymal stromal cells since the effects of MSCs are not multipotency. Compared to embryonic stem cells, MSCs can be obtained with invasive or minimally invasive procedures. The source of stem cells is abundance in adipose tissues, but limited in bone marrow (approximately 0.42%). However, the finding of adult stem cells might solve the ethical issues that are caused by using embryonic stem cell. Thus, MSCs are attractive cell types in tissue engineering and regenerative medicine [10]. Skeletal stem cells (SSCs) is another important stem cell type, which has been investigated in 2018 by Charles K.F Chan et al. SSCs isolated from fetal and adult bone can exhibit self-renewing and multipotent differentiation ability as other types of stem cell [2]. Comparative studies in BMSCs, UCSCs, and ADSCs suggested that they share common phenotype among 22 cell surface markers and differentiation potential; however, they noticeably differ in global gene expression patterns, proliferation rate, and clinical outcome [11,12]. Based on reported publications, Loubna Mazini et al. pointed that ADSCs are safer and more efficient in auto-immune diseases, UCSCs are mostly used in hematopoietic diseases, whereas BMSCs can be applied in immunosuppressive activity, hematopoietic self-renewal, and cancers [10]. The authors also reported that ADSCs were safe and efficient for systemic lupus erythematosus, systemic sclerosis, scleroderma, and Crohn’s diseases with no serious adverse effects being reported for short or long-time follow-up. BMSCs and UCSCs were limited for the small sample size and short-term follow-up of autoimmune disease’s treatment. Besides, the infusion of BMSCs was successful in improving the overall survival in most hematopoietic engraftment patients without any side-effects. Similar results were also reported with UCSCs after HSCs transplantation. BMSCs have been most commonly used among other stem cells; however, BMSCs characteristics, such as their differentiation potential, quantity, and lifespan, reduced with the age of the donors [10].

**Figure 1:**
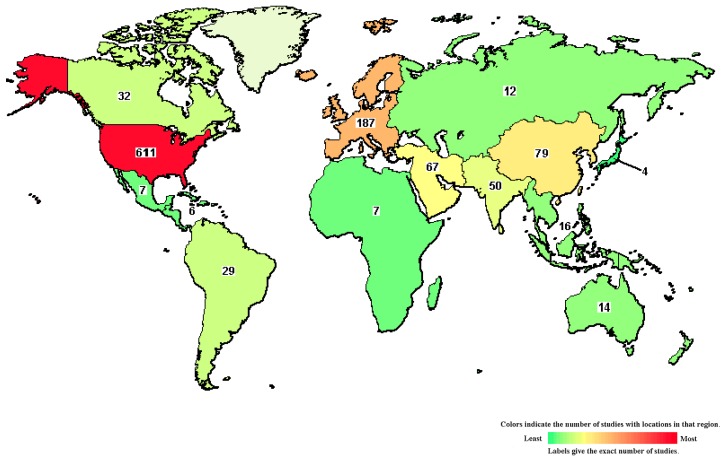
Map of clinical trials on bone marrow stem cells in the world [27]. North America comprises the highest number of trials, followed by Europe, the Middle East, and East Asia.

BMSCs are widely used in clinical applications. Friedenstein et al. first isolated adult non-hematopoietic stem cells from the bone marrow and transplant into patients [13]. Over a decade, BMSCs applications have been rapidly increasing. There are 5185 studies on “stem cells”, 1102 studies on “bone marrow stem cells”, and 759 studies on “mesenchymal stem cells” were registered in U.S National Library of Medicine database, according to clinicaltrials.gov (June 2019). Among “bone marrow stem cells” studies, most research has been completed phase I (safety) and phase II (efficacy) by using both allogeneic and autologous BMSCs. Interestingly, there are 95 studies on phase III and 20 studies on phase IV, which indicates the high potency of BMSCs in clinical uses compare to other stem cell types. Table 1 summarizes the number of trials. There are numerous completed trials conducted worldwide, however, mostly in North America with 630 studies (57.2%), followed by Europe 187 studies (16.9%) and the Middle East and East Asian countries (Figure 1). BMSCs are potential in the treatment of various diseases, consisting of muscle, bone and cartilage diseases, blood-related diseases, behavioral, and mental disorders, immune system diseases, nervous system diseases, viral/infective diseases, cancers, wounds, and injuries (available online in https://clinicals.gov). The phase III/IV trials were completed in the cardiovascular diseases, acute leukemia, rheumatoid arthritis, and diabetes mellitus.

Studies on BMSCs application provided promising results in many diseases. However, some studies showed varying outcomes, which might be caused by donor variability, cell preparation processes, and lack of standardization prior to transplantation [15]. The number of stem cells is limited in bone marrow (0.42%) when compared to adipose tissue and umbilical cord, thus the expansion and cultivation of BMSCs are required to obtain an efficient number of cells for treatment [16]. However, the expansion process highly contributes to genetic instability, senescence, and transformation issues [9]. In general, in-vitro expansion cells reduce the genetic stability of native BMSCs [9,17]. In vitro expansion reduces DNA polymerase and repair capacity, thus resulting in DNA damage accumulation [9]. Yi et al. developed and optimized GMP compatible culture protocol, which is suitable for producing BMSCs for clinical uses. Clinical-grade BMSCs are stable in terms of genomic integrity [18]. Even that, manufacturing systems hardly meet a standard requirement because of the differences between donors, harvesting methods, devices, and cultivation conditions [19]. 

The processes of harvesting BMSCs are invasive when compared to adipose stem cells. The donors often receive anesthesia during the procedure, resulting in serious risks (stiff or sore and tired) [20,21]. The recover capacity varies from several days to weeks. After transplantation, medications stimulate the mobilization of stem cells into the bloodstream [22]. However, medication can cause bone and muscle aches, headaches, fatigue, nausea, vomiting, and sleepless in patients. In fact, the massive number of donor cells die within hours or days after transplantation [23]. Steroids, interferon *γ* and in-vivo depletion of NK cells weaken the immune system, thus reducing the rejection of the donor cells and interaction donor’s immune cells with the recipient’s healthy cells. Besides, gene editing was also been exploited to avoid the unwanted responses of the immune system [24,25]. Autologous BMSCs transplantation causes no risk that is related to the immune system, graft failure, and treatment-related mortality, where all stem cells will be transplanted back to each patient, whereas allogeneic BMSCs transplantation is involved in the development of skin rash, diarrhea, abdominal pain, and hepatitis. However, autologous transplants could result in increased of risk for tumor formation. Autologous BMSCs transplantation is usually preferred for young patients with normal conditions in an effort to reduce the risk for toxicity and graft-versus-host disease that is associated with allogeneic therapy. The allogenic BMSCs therapy is more effectively and commonly treatment in elderly patients, ≥65 years of age who have decrease in response of immune system [26]. 

In conclusion, the current literature provides separately and inadequacy on BMSCs processing, transplantation methods, and clinical applications. Therefore, this manuscript has summarized the understanding of the research and clinical uses of BMSCs for five years (2014–2019) by searching related keywords in PubMed, google scholar, Elsevier, MDPI database, except for some major references. This manuscript showed the updated information of BMSCs on characteristics, isolation, expansion culture, differentiation potential, and application.

## 2. Characteristics of BMSCs

Bone marrow stem cells are known as non-hematopoietic stem cells (HSCs) that are located in the medullary stroma of bone marrow. BMSCs firstly discovered by Friedenstein et al. in 1976 and named as clonogenic fibroblast precursor cells (CFU-F) [28]. BMSCs have been used for tissue engineering and regenerative medicines [29]. However, BMSCs represent very low in bone marrow tissue, which ranges from 1/10,000 to 1/100,000. During standard culture, BMSCs can amplify 500-fold higher in 50 passages [30]. BMSCs population are heterogeneous [31]. The BMSCs’s characteristics are highly associated with the ages and/or pathological conditions of the donors [32]. The number of BMSCs and their differentiation ability decrease by aging, which is the result of DNA modification and transcriptional changes. Adipogenic, chondrogenic, and osteogenic differentiation capacity of murine BMSCs were decreased by the age of donor animals. Supported to the impact of aging, Olivia et al. showed old BMSCs suffered from reduced chondrogenic, adipogenic potential and impaired expansion properties [33]. Those findings indicated the donor’s age factor in cell-based therapies for older patients. Remarkably, BMSCs from old mice were much higher in terms of the presence of certain cellular senescence markers, such as DNA double-strand break marker γ-H_2_AX and DNA damage checkpoint response mediator 53BP1 than from young mice. Additionally, young BMSCs can increase the osteogenic activities and migration in mice. Transplantation young BMSCs can also extend life span when compared to non-transplantation and old BMSCs transplantation group [34]. Similarly, Stolzing et al. had shown age-related changes in BMSCs, consisting of stem cell number, marker phenotype, proliferation, differentiation potential, senescence and apoptosis induction, and stress level markers [35]. The authors reported the lower number of adherent cells being isolated from bone marrow, increase senescence and apoptosis marked by β-galactosidase positive cells, p53 and p21 expression during cultivation, higher ROS level in “aged” BMSCs when compared to “young” MSCs. Stem cells that were isolated from elders had a low rate of proliferation and differentiation ability into osteoblasts, whereas they increase the expression of apoptosis markers and SA-β-gal positive cells (an indicator of the senescence cells) [31]. The potential of transmitting diseases from the donor to recipient should be carefully considered, such as pathogens (bacteria, viruses, fungi, parasites), congenital disorders, autoimmune diseases, and malignancies [36,37]. Interestingly, these transmittable diseases tend to increase in prevalence with increasing donor age. Viruses like HIV type I and II, hepatitis B, C, CMV, leukemia-associated human T-lymphotropic virus I and II are most frequent in blood and stem cell products [37]. However, the risk of transmission of these viruses is quite low by current screening methods. Vaccination with live vaccines should be limited during the last two weeks of donation. Theoretically, all of the congenital diseases originating from bone marrow cells could be transmittable and should be considered as contraindications for stem cell donation. A patient died after receiving bone marrow transplantation from a donor with Gaucher’s disease [38]. While considering the immunodeficiency of recipient after conditioning, the malignant cells might engraft or metastasize leading to disease in recipient. Thus, clearly, the systematic evaluation of donor status becomes a necessity and mandatory, especially in elder donors. 

BMSCs secrete a variety of endocrine factors, which related to fibrosis, proliferation, apoptosis, chemotaxis, immunomodulatory, and angiogenesis processes in damaged tissues. By those effects, BMSCs can stimulate the recovery of the damaged area, the response of the immune system and protect other cells from apoptosis [31]. Table 2 summarizes the list of endocrine factors. 

Literature data on the effects of cell culture procedures, individual donors, and clinical conditions are still scarce. Recently, a study has shown significant increase the expression of proteins that are associated with angiogenic signaling pathways under ischemic conditions [39]. Similarly, Salwa et al. have just reported the impact of isolation and culture conditions on the stemness and differentiation potential of BMSCs [40]. The authors investigated cells that were expanded in human serum and FGF2 condition expressed higher osteogenic markers; cell expanded in lysate plasma showed significant upregulation of IL-1α and IL-1β expression. Lysate plasma condition enhanced more stem-like phenotype, delayed differentiation, and changed the inflammatory gene expressions. Moreover, allogeneic HSCs located in bone marrow often cause graft-vs-host disease (GVHD) when the donor’s immune cells attack the healthy cell in patients [41]. The secreted factors and functional of BMSCs are altered, due to the donor’s health, age, and exposure to environmental stresses [42].

An important characteristic of MSCs is the immunoregulatory capacity and elicit immunosuppressive effects. DeMiguel et al. showed these effects in a number of situations, including decreased expression of class II Major Histocompatibility Complex (MHC-II) and costimulatory molecules via direct cell-to-cell interaction and soluble factor secretion. MSCs are capable of inhibiting the cell proliferation of T cells, B-cells, natural killer cells (NK), and dendritic cells. Besides, MSCs can block cytokine secretion and cytotoxicity of T can NK cells, B cell maturation, dendritic cells maturation, and activation, as well as antigen production. Thus, it is clear that MSCs exhibit their immunomodulation activities [43]. 

In general, MSCs can be obtained by a different method of isolation, cultivation, and characterization, thus BMSCs may vary the expression of cell surface markers. ISCT released the gold standards of BMSCs to classify them among other bone marrow cells [8]. Undifferentiated BMSCs, as well as other MSCs, which are plastic adherent cells, show positive expression of CD105, CD73, CD90, and lack the expression of CD45, CD34, CD14 and CD11b, CD79α, and CD19 at least in the standard culture. Currently, the STRO-1 marker, which is not involved in ISCT criteria, is a defined marker for mesenchymal stem cells from bone marrow. STRO-1 highly expresses in fresh and young BMSCs and reduces during expansion culture, thus positive STRO-1 BMSCs are thought to be suitable for clinical application [29]. The higher expression of CD271 and PDGFRα is associated with the skeletal differentiation capacity of BMSCs. 

Besides the expression of surface markers, BMSCs can be characterized by self-renewability and multi-lineage differentiation capacity. In vitro, BMSCs can differentiate into adipocytes, osteoblasts, chondroblasts, neural, myocyte, and epithelial cells by the supplementation of stimulating factors to the cultures. However, the potential for adipocytes, osteoblasts and chondroblasts differentiation of BMSCs subpopulations should be fully characterized [29,44]. The culture process of BMSCs causes heterogeneity in differentiation potential. Additionally, antibiotics and serum that are added in the culture medium can affect the phenotype and differentiation potential of BMSCs [29]. 

## 3. Processes in Isolation of BMSCs

It is clear that both unfractionated bone marrow or specific subpopulation of mesenchymal stem cells can be applied in stem cell therapy [65,66,67,68]. The isolation of BMSCs has been developed and used to characterize mesenchymal stem cells in mouse, rabbit, dog, pig, rat, and human [69,70,71,72,73,74]. Hematopoietic stem cells (HSCs) and mesenchymal stem cells, which localized together in the bone marrow, are most commonly used in clinical application. The isolation and enrichment strategies and characterization of BMSCs are based on their biological characteristics, which consist of plastic-adhesion, surface markers expression, and multiple lineage differentiation capacities [72,75,76]. Traditionally, BMSCs will be collected by specific bone marrow aspiration and selected as plastic adhesion cells in vitro. This method is simple and convenient when compared to other stem cells; for instance, adipose stem cell isolation requires enzymes to digest adipose tissues. However, it is not enough to distinguish between BMSCs and other bone marrow subpopulations, such as endothelial cells, pericyte, leukocyte, and hematopoietic stem cells [16]. Thus, other isolation methods have been accessed including a density gradient centrifugation method, named as Ficoll method [77], as well as advanced cell sorting methods, such as the flow cytometry-based sorting method [72,78]. Additionally, MACS technology is also fast and efficient for stem cell separation. The flow cytometry and MACS technologies are both based on the expression of cell surface markers. For MACS, magnetic labeling using CD271 used a marker for isolation of MSCs from bone marrow and lipoaspirate. MACS microbeads, columns, and automated separators are available to apply in stem cell separation. ISCT published the guideline to the cell surface markers isolation BMSCs, which is suitable for clinical application [8]. Human BMSCs will be sorted with positive markers CD105, CD73, CD90 and negative markers CD45 (hematopoietic cell marker), CD34 (endothelial cell marker), and HLA-DR (leukocyte marker). Besides, other strategies that are based on the enrichment of SRO-1, CD146, SSEA-4, CD271, and MSC antigen-1 to purify BMSCs [29,79,80]. Samsonraj R.M et al. listed the surface antigen expressed in BMSCs [29]. The combination of CD49a, PDFGRα/β, EGFR, IGFR, and STRO-3 markers was also reviewed as an isolation criterion of BMSCs. The authors demonstrated that STRO-1 and PDGFRα makers supported the selection of BMSCs, which showed higher bone regeneration potential. While Feng-Juan Lv et al. opposed the phenotype and expression of stem cell surface markers are varied by the expansion culture condition [80]. Therefore, it is a clinical, legal, ethical obligation to follow GMP guidelines in the isolation of BMSCs, where the isolation and culture of BMSCs will be done in the automated systems [81,82,83]. The GMP-compliant isolation and expansion procedure of BMSCs will be discussed in the following section. 

## 4. Processes Involved in the Culture of BMSCs

It is necessary to amplify BMSCs in vitro before clinical application to get a sufficient number of cells for the therapeutic dose due to the low number of stem cells in bone marrow (0.001–0.01% total nucleated cells and 0.42% plastic adherent cells) [16,84]. In general, the risks of use BMSCs are strongly associated with culture conditions. Therefore, large-scale BMSCs production must adhere to the requirement of GMP standardization. Ayesha Aijaz et al. generally discussed bio-manufacturing for clinical cell therapies [85]. The authors demonstrated four parameters that should be considered: quality, cost, scalability, and sustainability in the cell therapy products manufacturing. 

### 4.1. Culture Expansion Conditions

In Europe, the European Medicines Agency approved mesenchymal stem cells as advanced therapy medicinal products (ATMPs) from 2007 [86]. In the United States, BMSCs must comply with Current Good Tissue Practice (CGTP) guidelines. Accordingly, the BMSCs must be produced in the GMP-grade control to ensure reproducibility, safety, and efficiency of stem cell therapy. During the cultivation of BMSCs, sources and harvest methods, cell seeding plate and density, proliferation rate, and culture medium should be followed and then validated in the variety of cell expansion systems [87]. Generally, BMSCs can be harvested by either invasive or non-invasive methods. The noninvasive method relies on salvaging the large bones of a freshly slaughtered animal, whereas invasive methods can be applied on a live animal, followed by density gradient centrifugation. The noninvasive method was found to be much less cumbersome, however, it cannot be used to harvest BMSCs in humans. Invasive methods consist of perfusion or aspiration method, where the aspiration method yielded significantly higher than the perfusion method. However, the perfusion method is simple and safe with minimal contamination and it reduces the risk of acute graft versus host disease in allogeneic bone marrow transplantation [88]. Time culturing decreased the proliferation rate and multipotent differentiation potential of stem cells [87]. Moreover, the age of donors also influenced the proliferation rate of cultured BMSCs [35]. In terms of culture medium, Dulbecco’s minimal essential medium (DMEM) supplied with 10% of approved fetal calf serum (FCS) was basically used in the culture of BMSCs to minimize the variety of batch to batch variations of FCS and reduce the risk of allergies related to xenogeneic proteins to patients [89,90]. Plasma temp platelet lysate and platelet-rich plasma (2–8%) or growth factors, such as TGF-β, FGF2, or PGFEP, can be supplied in the media instead of serum. Those media efficiently maintain the morphology and multi-lineage differentiation capacity of the BMSCs population [90]. Many studies pointed to FGFs as major players in seft-renewing proliferation, cellular senescence, and aging. Additionally, FGF-2 not only promotes cell growth level, but also maintains specific stem cell markers and improves chondrogenic differentiation [91]. Therefore, FGF-2 is frequently added during the pre-selection of MSCs subpopulation. However, FGFs are key challenges for the cost-effective expansion of BMSCs. Researchers had been finding other components instead of FGFs. The animal component-free and serum-free media have been developed and marketed, which is convenient for the culture of human mesenchymal stem cells, such as MesenCult™-ACF Plus Medium (STEMCELL Technologies), CTS StemPro MSC SFM, and StemPro MSC SFM XenoFree (ThermoFisher Scientific, Waltham, MA USA 02451) or Pro293a (Lonza, Basel, Switzerland). In comparison to the traditional culture medium, human MSCs that were expanded in serum-free and xeno-free media showed better expansion, multipotentiality, and serve as suitable for the production of large scale, functionally competent MSCs for clinical applications [92]. In 2012, Natalie Fekete et al. validated five different GMP-compliant protocols for the isolation and expansion of BMSCs consisted of a single-step and four two-step protocols [81]. Single-step protocol was primary culture with one circle of seeding and harvesting steps, while two-step protocol contained two circles. The authors reported that the single-step or two-step protocols can be suitable to produce at least single therapeutic dose. The single-step protocol used short-time cultivation, whereas the two-step protocol was used to obtain a much higher amount of BMSCs and to reduce the costs for expansion. However, the authors also mentioned the long-term culture of BMSCs leads to a change in the genotype as well as differentiation characteristics of BMSCs. Besides, Yi et al. developed and optimized GMP compatible culture protocol, which is suitable for producing BMSCs for clinical uses. The final products, which were produced from cells obtained at passage 12, must be satisfy all of these criteria: pathologic microbes, mycoplasma, cytopathic effect and hemadsorption, cell morphology, virus test, MSC marker analysis, and cell viability. Karen Bieback et al. also reported typical clinical protocols for the isolation and expansion of MSCs [93]. The authors described the essential processes and necessary quality assessment parameters performed, including ENTRY CONTROL (cell number, viability, sterility) -IN PROCESS CONTROL (cell number, viability, sterility, clonogenicity, immune phenotype) -RELEASE CONTROL (cell dose, viability, immune phenotype, microbial, endotoxin, mycoplasma test) -POTENCY ASSAYS (clonogenicity, trilineage differentiation, immunomodulation, hematopoiesis regulation). That method is an efficacious and economical option in clinical settings. The released parameters for MSCs manufacturing (Table 3).

### 4.2. Devices Using in Culture Expansion

Tissue culture treated flasks or dishes are important for traditional monolayer culture of mesenchymal stem cells; however, the traditional culture requires a class A cabinet to avoid a high risk of contamination with bacteria or fungi. Thus, closed and semi-automated/automated systems have been developed that fulfill GMP requirements [82,83,94]. The Quantum cell expansion system (Terumo BCT, Lakewood, CO, USA), a hollow fiber bioreactor system, has been commonly used in several investigations [82,83]. Those scale-up cell manufacturing systems overcame the conventional methods in terms of minimal media exposure, manufacturing time, anticontamination, and labor costs. Moreover, the Quantum cell expansion system is reliable, reproducible, and most economical to produce sufficient BMSCs for a clinical trial (single dose of 100 × 10^6^ cells/subject × 100 subjects) [83,94]. The authors described the full end-to-end protocol of the BMSCs manufacturing process, consisting of media preparation, set up the system, coating of fiber, loading sample, feeding, and harvesting. Additionally, the authors suggested using fibronectin, platelet lysate, and human plasma to coat the hollow of the quantum system when loading raw BMSCs [82].

### 4.3. Issues upon Culture Expansion

Lastly, the culture condition induced morphological, phenotypic, and genetic alterations of BMSCs, which Yang et al. investigated [95]. Long-term culture expansion reduces cellular proliferation, differentiation potential, homing, and immunomodulatory properties, while developing senescence and tumor generation [87]. The cultivation of BMSCs was interrupted at passages 25, where the cells might stop proliferation and go into senescence state [96]. Significantly, decreases in differentiation potentials were also observed due to passage [97]. Currently, several techniques are available to detect or quantify the genomic alteration, listed of conventional karyotype, Spectral Karyotype, Array-CGH (comparative genomic hybridization), virtual karyotype, FISH fluorescent in situ hybridization, microsatellite instability analysis, single nucleotide polymorphism array, gammaH2AX, telomere length by Southern, Sanger sequencing, NGS next-generation sequencing, the droplet digital PCR, micronuclei test, and comet assay. Additionally, it was also demonstrated that long-term cultures under hypoxic conditions prevent senescence, improve DNA damage response while maintaining differentiation potential. The authors determined that DMEM-based medium facilitated cell proliferation in the early passages, but reduced cell population doubling rate in the aged passages. It also caused a decrease in the expression of CD146, especially STRO-1, which was lost during in-vitro expansion. Besides, microscopic image demonstrated aging MSCs that were cultured with either medium formation increased morphological inhomogeneity and gene expression profile. Therefore, the differentiation potential of MSCs was also affected during culture period. MSCs are able to maintain adipogenic differentiation, whereas senescence significantly impaired osteogenic potential.

## 5. Processes Involved in the Differentiation of BMSCs

### 5.1. Differentiation Potentials of BMSCs and Regulation

BMSCs are well known for their multi-lineage differentiation potential into osteocytes, adipocytes, chondrocytes, and skeletal muscle [98,99,100]. In-vitro differentiation culture conditions generally regulate the differentiation capacity of BMSCs. The alkaline culture medium can stimulate the differentiation of BMSCs into chondrocytes [101]. Dexamethasone is required for osteogenic differentiation [102]. Bone morphogenetic proteins (BMPs) can induce BMSCs into osteocytes by regulating the expression of Runt-related transcription 2 (Runx2) [103]. Additionally, three-dimensional culture and azacytidine (an anticancer drug) can enhance the osteogenic and adipogenic differentiation ability of BMSCs [104]. Unbalanced bidirectional differentiation between adipocytes and osteocytes might relate to osteoporosis, which characterized by decreasing the number of osteoblast cells and increasing adipocyte cells. To date, the co-treatment of estrogen can promote osteogenic differentiation, while inhibiting the adipogenic differentiation of BMSCs in osteoporosis patients. Besides, 1,25 dihydroxy vitamin D3 inhibits PPARγ2, thus suppressing lipogenesis and adipogenic differentiation [99]. Receptor activator of nuclear factor κB ligand [105] signaling inhibits osteogenesis, but does not affect adipogenesis of BMSCs [106]. Jicheng Wang et al. reported the role of miRNAs in the osteogenic differentiation of BMSCs. The authors identified 17 miRNAs that were upregulated and five miRNAs were downregulated, which target various genes during osteoblast differentiation when compared to undifferentiated BMSCs [107]. These findings were consistent with the findings of Ying Chen et al. [108]. Moreover, a balanced differentiation of BMSCs also contributes to hematopoietic recovery after bone marrow transplantation [109]. In vivo, many factors, such as aging, obesity, irradiation, and chemotherapy, cause the alteration of the microenvironment, thus modulate differentiation capacity of transplanted BMSCs [109]. These factors regulate BMSCs differentiation by a series of signaling pathways, which results in the imbalance of adipogenic-osteogenic differentiation, leading to the accumulation of adipocytes and the attenuation of osteocytes at the implantation site.

### 5.2. Trans-Differentiate and De-Differentiate Potentials

Interestingly, BMSCs are able to trans-differentiate into other cell types and de-differentiate back to the undifferentiated state in response to extracellular signals [110]. BMSCs can be differentiated and dedifferentiate into neurons and cardiomyocytes, respectively [111,112]. Lui et al. succeeded in inducting of BMSCs into neurocytes and reverting neurocytes to de-differentiated BMSCs in-vitro by removing the extrinsic factors [112]. De-differentiated BMSCs showed increasing cell survival and differentiation potential, thus improving cognition function of brain damage in rats when compared to unmanipulated BMSCs. Yannarelli et al. also investigated partly cardiomyocytes differentiation potential of BMSCs. Besides, partially cardiomyocytes differentiated cells can revert to undifferentiated state of MSCs, and potential to extended differentiation via the regulation of pluripotency transcription factor OCT4 [111].

## 6. Processes in the Storage of BMSCs

### 6.1. In Short-Term Storage

The wide use of BMSCs as regenerative medicine in the clinic requires strictly controlled quality in the process and the final product. Storage and transportation significantly influence the viability and differentiation capacity of BMSCs preparation prior to transplantation. Several medium and temperature conditions, as well as the maximum time of storage, have been evaluated, which supports the safety and potency of stem cell therapy [113,114,115]. Mesenchymal stem cells should be stored in 0.9% saline for less than two hours. After four hours, the proliferation of stem cells rapidly reduces, and the cell viability decreases when compared to fresh BMSCs (>90% of the cell population). Longer storage leads to a significant reduction of self-renewal and differentiation capacity [113]. The authors report that temperature (4 °C or room temperature) did not affect the viability of stem cells. Most recently, the trehalose-based solution has been recommended for the storage of BMSCs at 4 °C for 72 h [115]. This trehalose solution enhanced the recovery rates, the self-renewable capacity of BMSCs with a consistent phenotype and genotype, as well as the differentiation properties of BMSCs when compared to Plasma-Lyte^®^ 148, HypoThermosol^®^ FRS, and Ringer’s solution that is commonly used in clinical research [115]. Currently, there are no specific transportation systems for stem cells in clinical applications. Tomoki Aoyama et al. evaluated both frozen and non-frozen transportation systems and then suggested the critical parameters that are essential to be considered during transportation of BMCs [116]. The authors demonstrated that the transported materials and cell products require satisfying some requirements, including leakproofness, sterility, temperature stabilization, shock resistance, gas stability, UV shielding, and monitoring. DMSO can be used as cryoprotectant, but it has some adverse effects on patients, such as nausea, headache, hypertension, and diarrhea. Therefore, the International Stem Cell Initiative recommends the use of liquid nitrogen (−196 °C) for frozen stem cell transportation. However, a significant loss of living cells and increase in the proportion of apoptotic and senescence cells regarding frozen transportation should be considered. In non-frozen cell transportation, the control of temperature (37 °C) and cell metabolism (added preservation solution) is compromised. Besides, non-frozen transportation methods are not convenient, because of the limitation of transportation package for long distance.

### 6.2. In Long-Term Storage

Stem cell banking was supposed to store a maximum number of samples with effective costs [114,117]. Cryovials or cryobags can be used in various sizes. Cryovial sizes vary from 2.0 to 4.5 cc, whereas the cryobag volumes mostly are bigger, from 25 to 60 cc. In addition, cryobags can be applied in closed automated systems, allowing for culture, selection, and storage procedures. However, cryovials are commonly used in an open system that requires sterility control during processing. BMSCs can be stored in liquid nitrogen (temperature always below −196 °C) for a long time (>1 year). Stem cell banking can hold 10,000 25-cc bags or 40,000 4-cc vials. Most importantly, the stem cell banking must be under the quality control at each time point to provide a high quantity and quality of cryopreserved samples.

## 7. Potency Analysis for BMSCs Production

It is important to evaluate the quality of stem cell products for clinical applications. The identification of relevant and robust potency assays is not only a regulatory requirement, but it is also the standard for producing and delivering a product that is consistent, safe, and ultimately an effective therapy. It is no doubt that the cells that were collected from different tissue sources and expansion methods showed the same morphologic, cell surface markers, and differentiation characteristics (minimal criteria for MSCs defined by ISCT), but still had major differences in their biologic and functional actions. However, despite extensive ongoing researches regarding the use of BMSCs, there is no consensus on any potency assay for MSCs, because stem cells have a complex and/or not fully characterized mechanism of action. Currently, a single bioassay approach and multiple complementary assays approach have been investigated [118,119,120,121]. For instance, Lee et al. demonstrated that the flow cytometry-based quantitative IDO assay correlates with MSC’s inhibition of T cell proliferation and results in phase I of autologous MSC- based clinical trial for the treatment of osteoarthritis [120]. Chinnadurai et al. developed MSC secretome analysis (29 cytokines) and a quantitative RNA-based array (~40 genes) for genes specific to immunomodulatory and homing properties of MSCs [121]. Even then, a single bioassay method might not provide an adequate measure of the potency for a cellular product, such as BMSCs as multiple complementary assays. To date, the issue of developing appropriate potency assay for BMSCs remains very challenging. The test must include appropriate controls and standard materials; validate to meet the requirements, including accuracy, precision, repeatability, specificity, linearity and range, system suitability, and robustness; and, be convenient to perform as a quality control test.

## 8. Processes Involved in the Application of BMSCs

**Figure 2:**
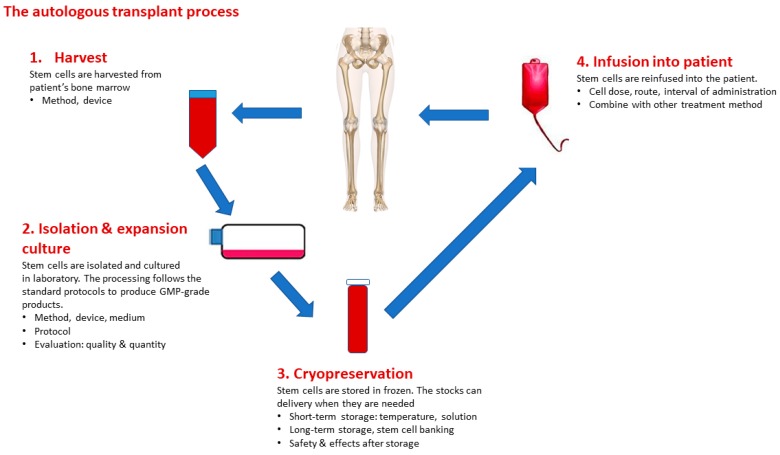
The process of bone marrow mesenchymal stem cell transplantation.

Human bone marrow stem cells have been investigated as potential therapeutic strategies for various human diseases, especially in the field of regeneration medicine [122]. BMSCs have the ability to self-renew, multi-lineage differentiation, migration, anti-inflammatory, and immunomodulation, which may, together, support therapeutic effects in clinical applications. It is clear that both autologous and allogeneic BMSCs have been used in stem cell therapy. Autologous BMSCs can bring lower risks that are related to the immune systems, but a higher risk of tumor formation. Several studies have observed potential adverse effects of MSCs, including tumor growth, metastasis, and transformation into cancer cells [123,124,125]. Specifically, human pulmonary MSCs exhibited high proliferative capacity with unbalanced chromosomal rearrangements [126]. These adverse effects may be explained by a heterogeneous MSCs population used in experiments that contaminated “abnormal cells”—the cells initially grew slowly and then transformed into cancer cells. In fact, the impact of the administration of MSC on tumor growth is controversial. In vitro and in vivo studies have reported stimulation of tumor growth by providing supportive stroma, creating a permissive environment and/or reducing immune rejection of tumor cells [127,128]. A further risk that is associated with MSC expansion in vitro is senescence, which only occurs after long-term passage in culture, and subsequent differentiation into tumor cells after in vivo transplantation, has been shown in rodents [129]. Although concerns that MSCs might transform into tumor cells still exits, there is general agreement that BMSCs can be safe with no risk of malignant transformation, and so far no cancer has been yet reported in clinical trials for autologous cell therapy, thus making MSCs suitable for use in trials [96]. Constantly, in allogeneic cells transplantation, immune rejection of allogeneic cells might prevent the development of tumors in vivo. Allogenic BMSCs transplantation usually uses in the treatment in elderly patients, who have suppression of immune responses. Cell death or apoptosis might be observed within a few days after stem cell engraftment, due to harsh environmental conditions, anoikis, and inflammation [130]. Chronic inflammation inhibits the recruitment and survival of implanted cells, promotes the generation of ROS, thereby inducing apoptosis. Therefore, methods for reducing apoptosis and enhancing cell adhesion have been approached to improve the survival of the cells at the transplanted sites. The authors demonstrated pretreatment with cytokines, growth factors, and antiapoptotic molecules, genetic modification, and preconditioning can be useful for improving the survival rate of transfected cells. Besides, hypoxia or the three-dimensional cell culture system were also approached to improve the immunomodulatory properties of human MSCs. Figure 2 describes the process of autologous BMSCs transplantation on human.

### 8.1. BMSCs Involved in the Treatment of Immune Disorder Diseases

BMSCs have been investigated in the treatment of immune disorder diseases, including GVHD, type I diabetes, systemic lupus erythematosus (SLE), and rheumatoid arthritis (RA) [131]. By secreting soluble factors, MSCs can inhibit immune cell migration, proliferation, differentiation, and activation, which result in the suppression of immune responses. GVHD is a severe inflammatory condition that is caused by the attacking of donor T cells to the immune system of the recipient after allogenic HSCs transplantation. To date, corticoids are commonly used in the initial treatment of GVHD and BMSCs as an alternative option to treat in corticoids-resistant patients. FDA approved a commercialized product, named Prochymal (Osiris Therapeutics Inc., Columbia, MD, USA), which contains BMSCs to treat GVHD, specifically in pediatric patients with serious corticoids resistance [132,133,134]. Recently, a meta-analysis has shown that BMSCs can significantly reduce the incidence rate of chronic GVHD but not acute GVHD in patients. To prevent GVHD, it is important to use BMSCs after HSCs implant to minimize the side effects of stem cell therapy [135]. Besides, BMSCs also applied to treat chronic inflammatory diseases [136,137]. A comparative study showed that the systemic infusion of BMSCs significantly improved symptoms of RA in mice [138]. BMSCs reduced bone erosions, synovitis and articular destruction, levels of TNF-α and IL-1β in serum and joint, which results in an improvement of inflammation state in RA mice. BMSCs are considered to be the best stem cells for the treatment of RA when compared to UCSCs and exfoliated deciduous teeth stem cells [138]. In addition, a randomized, triple-blind trial showed the improvement of Western Ontario and McMaster Universities Arthritis Index (WOMAC), visual analog scale (VAS), time to jelling, pain-free walking distance, and standing time in 30 RA patients treated with BMSCs [139]. However, the trial failed to prolong the therapeutic effects of BMSCs beyond 12 months. In SLE patients, BMSCs are safe and they contribute to improving the SLE Disease Activity Index (SLEDAI) score, blood parameters, and overall survival rate [140,141,142]. JAK/STAT, PTEN/AKT, p53/p21, and Wnt/β-catenin signaling pathways were investigated in mediating BMSC senescence and apoptosis, which might contribute to the development of SLE [143,144,145,146]. On the other hand, the infusion of BMSCs suppressed helper T-cell development, thus attenuating immune responses and inflammatory damage [147]. Type I and type II diabetes have been the targets of BMSCs therapy [148,149,150,151]. There are several completed and in processing clinical trials registered in the database of U.S National Library of Medicine that used BMSCs in phase I to phase III clinical trials. After nine months of BMSCs intravenously transfusion, diabetic patients are free from insulin injections without any side effects [151]. In addition, a randomized trial in 21 patients who underwent BMSCs infusion of a superior pancreaticoduodenal artery or splenic artery significantly reduced the insulin requirement dose, but did not show the improvement of insulin sensitivity [150]. Intravenous BMSCs failed to show the improvement of stem cell therapy in this trial. In addition, the evidence of BMSCs that may differentiate into β-cells is unclear. However, BMSCs can enhance the vascular generation, endothelial repair, and anti-inflammation through paracrine activity [149]. MSC-derived exosomes and microvesicles are important mediators of the paracrine activity. These extracellular vesicles can regulate host cells and surrounding microenvironment and improve the disease outcomes, which consist of mRNAs, microRNAs, cytokines, and growth factors [152,153,154,155]. In addition, the extracellular vesicles have been recognized as a drug delivery platform, which brings chemicals or drugs to the target cells [156]. 

### 8.2. BMSCs Involved in the Treatment of Neurodegenerative Diseases

BMSCs have been widely applied to various neurodegenerative diseases, which consist of multiple sclerosis, amyotrophic lateral sclerosis (ALS), Parkinson’s disease (PD), and Alzheimer’s disease (AD). Intrathecal injection of expanded BMSCs are safe and enhanced the improvement parameters in advanced multiple sclerosis in the 10 patients studied for 3–6 months treatment [157]. Another study also agreed that intravenously delivered MSCs limit neuronal damage by indirectly regulating immune systems and regenerating the neurons [158]. Research group [159] recently published the protocol for assessing the safety and efficacy of a single intravenous dose of BMSCs for multiple sclerosis, which is the guideline for studying BMSCs in humans [159]. BMSCs transplantation has shown self-renewable and neural protective properties in ALS patients. BMSCs are safe in phase I/IIa clinical trial in ALS patients [160,161,162]. Intravenous and intrathecal injection of BMSCs showed a reduction of the ALS Functional Rating Scale and Forced Vital Capacity percentage in all of the treated patients [161]. In PD, BMSCs have the ability to regenerate damaged neurons [163,164]. Moreover, BMSCs can improve the dopamine transporter binding activity, which results in the recovery and protection of dopaminergic neurons [165]. Several clinical trials are conducted while using BMSCs on PD patients (NCT02611167, NCT01446614, NCT00976430). The completed trials showed improvement in PD patients without any side effects after 36 months of BMSCs transplantation [163]. Human BMSCs reduced IL-1, IL-2, TNF-α, and IFN-γ in serum and oxidative stress, which showed the anti-inflammation potential and regulation of the expression of amyloid β1–42 genes and, thus, improved the symptoms of AD [166]. The improvement effects were seen in rat intracerebral hemorrhage after the administration of BMSCs to induce anti-inflammation and neurogenesis properties [167]. However, the therapeutic effects of BMSCs are still being investigated, and the future results may provide better evidence to widely apply BMSCs in the clinic. 

### 8.3. BMSCs Involved in the Treatment of other Diseases

Lastly, BMSCs have shown potential on the treatment of osteoarthritis (OA), a degenerative disease of bone [168,169,170,171,172,173,174]. In a meta-analysis study, BMSCs improved knee joint cartilage degeneration, joint damage, and significantly reduced pain [168]. Six clinical trials showed an improvement of patient-reported outcomes, that support stem cell injection in knee osteoarthritis [175]. A systematic review of 659 studies showed a significant reduction of knee pain, self-reported physical function, and cartilage quality evidence to use in rehabilitation for OA patients [176]. Besides, BMSCs is also intended in the treatment of sports injuries [177,178]. 

**Figure 3:**
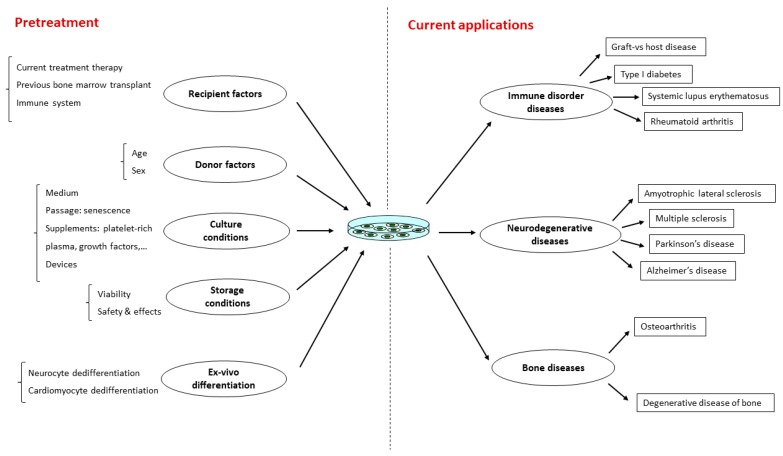
Summarize the current clinical applications of bone marrow mesenchymal stem cells.

Overall, BMSCs are popular adult stem cells and they have long been therapeutically applied in various diseases. This review summarized the preclinical and clinical studies of BMSCs in Table 4 and Figure 3. The number and size of trials are still limited; therefore, it is necessary to conduct wide studies that will support the use of BMSCs in clinical application. 

## 9. Conclusions

Research on the BMSCs has been gradually increasing over recent years. BMSCs became the most commonly mesenchymal stem cells used in the clinic as regenerative medicine. In-vitro studies on isolation, culture and differentiation, and storage processes of BMSCs support the wider application of BMSCs in clinical practice. As the number of BMSCs is very low in bone marrow, the expansion culture is mandatory for producing clinical-grade production. Under the long-term expansion, BMSCs may undergo senescence and transformation that contribute to the development of tumors and metastases. Therefore, the processing of BMSCs must satisfy the good manufacturing practice requirement to ensure the quantity and quality of BMSCs, which are strongly associated with the safety and outcome of BMSCs therapy. Most of the preclinical and clinical trials have shown promising results of BMSCs on the treatments of various diseases with few adverse effects during follow-up periods. Currently, BMSCs therapy has been used in the treatment of osteoarthritis, immune disorder diseases, neurodegenerative diseases, and sporty injuries. Autologous and allogeneic BMSCs both can be used in clinical trials and exhibit positive results. However, the number of subjects in clinical trials and conditions of treatment is limited. Therefore, it is necessary to conduct more randomized, controlled clinical trials to find the best standards for BMSC therapy to evaluate the safety and benefits of each application. 

## 10. Future Directions

It is now clear that stem cell therapy has gained interest in recent years. Preclinical and clinical studies have been conducted to provide evidence for using stem cells, especially BMSCs in the clinic. There are several issues that need to be further investigated before applying stem cells. Recent studies have shown BMSCs can stimulate lung tumor growth and metastasis [194,195,196]. Few adverse effects were also reported by the treatment of BMSCs. Moreover, the number of human trials is limited in phase I and phase II; and, lacks phase III and IV trials. Therefore, further studies may focus on the safety, molecular mechanism, and standardization of the stem cell preparation processes as well as shipping and clinical application. The safety of BMSCs therapy still needs to be carefully investigated by expanding in terms of a number of studies, and the size of subjects in phase I/II. Cells harvesting, expansion processes, and quality control must follow a standard protocol or GMP guideline. Diseases and patient conditions must optimize cell dose, route, and interval of BMSCs administration. In addition, understanding the molecular mechanism in the homeostasis, regulation tissue repair, modulation of immune responses, and differentiation of BMSCs may support new methods for improving the efficacy of trials. Finally, a combination of BMSCs therapy and other methods should be combined that may provide promising outcomes of BMSCs applications. BMSC therapy combined with drug treatment for ischemic stroke is potentially a feasible and efficient therapeutic approach. They exert synergistic effects in different ways, including enhancing stem cell migration and survival, promoting endogenous stem cell proliferation, reducing apoptosis, and angiogenesis. For instance, sodium ferulate accelerates BMSC migration toward the ischemic zone in a rat model [197]; valproate- or lithium-pretreated BMSCs stimulate cell migration and targeting ability and promote functional recovery [198]; erythropoietin acts synergistically with MSCs to potentiate neurogenesis [199]. This combination approach should be further evaluated in clinical trials.

## Figures and Tables

**Table 1:** The number of bone marrow stem cells trials classified by the phase [14].

Phase	Number of Studies	Note
Early phase I	16	Testing in non-human subjects
Phase I	412	Testing safety in 20–100 normal healthy volunteers
Phase II	610	Determination of therapeutic dose in 100–300 patients
Phase III	95	Determination of therapeutic dose, safety, and efficiency in 300–3000 patients
Phase IV	20	Testing the long-term effects
Not applicable	108	-

**Table 2:** Endocrine factors secreted by bone marrow mesenchymal stem cells.

Secreted Factors	Function
Basic fibroblast growth factor (bFGF) [45,46]	Cell survival, proliferation, and differentiation
Insulin-like growth factor (IGF) [47]	
Secreted frizzled-related protein-1 (SFRP1) [48]	
Secreted frizzled-related protein-2 (SFRP2) [49]	
Stanniocalcin-1 (STC-1) [50]	
Transforming growth factor β (TGF-β) [46]	
miR-10b-5p, miR-22-3p, miR-191, miR-222, miR-21, let-7a [51]	
Metalloproteinase-1 (MMP1) [46]	Remodeling of extracellular matrix
Metalloproteinase 9 (MMP9) [52]	
Plasminogen activator (PA) [46]	
Tumor necrosis factor-α (TNF-α) [46]	
Angiopoietins (ANGs) [53]	Angiogenesis
Fibroblast growth factor-2 (FGF-2) [45]	
Transforming growth factor β (TGF-β) [46]	
Vascular endothelial growth factor (VEGF) [54]	
miR-132 [55]	
miR-222, miR-21, let-7f [51]	
Hepatocyte growth factor (HGF) [56]	Immunomodulatory
Human leukocyte antigen G5 (HLA-G5) [57]	
Indoleamine 2,3-dioxygenase (IDO) [58,59]	
Inducible nitric oxide synthase (iNOS) [60]	
Interleukin-6 (IL-6) [61]	
Interleukin-10 (IL-10) [62]	
Leukemia inhibitory factor (LIF) [63]	
Prostaglandin E2 (PGE2) [64]	
Transforming growth factor β (TGF-β) [46,56]	
miR-143-3p [51]	

**Table 3:** The released parameters for mesenchymal stem cells (MSCs) manufacturing.

Characteristics	Parameters	Requirements
ENTRY CONTROL	Cell number	>2 mL sample
	Viability	>90%
	Sterility	Positive
IN PROCESS CONTROL	Cell number	5–10 × 10^8^ MSCs
	Viability	>90%
	Sterility	Positive
	Clonogenicity	1 to 5 MSCs/cm^2^
	Immune phenotype	CD105, CD73, and CD90 (>95% total cells). CD34, CD45, CD14 or CD11b, CD79α or CD19 and HLA-DR (≤2%).
RELEASE CONTROL	Cell dose	More than 5 × 106 MSCs/kg body weight of the recipient
	Viability	>90%
	Immune phenotype	CD105, CD73, and CD90 (>95% total cells). CD34, CD45, CD14 or CD11b, CD79α or CD19 and HLA-DR (≤2%).
	Microbial	Negative
	Endotoxin	Negative
	Mycoplasma test	<50 CFU/ml
POTENCY ASSAY	Clonogenicity	1 to 5 MSCs/cm^2^
	Trilineage differentiation	Positive
	Immunomodulation	Positive
	Hematopoiesis regulation	Positive

**Table 4:** Preclinical studies and clinical trials of bone marrow stem cells (BMSCs) applications.

Diseases	Pre-Clinical Studies	Clinical Trials	Routine Treatment	Effect of BMSCs Therapy	Autologous or Allogenous	Ref.
Graft versus host disease	-	Phase II/III	Infusion	Improve the overall survival rate	Allogenous	[179]
	-	Phase I	Injected intravenously	No acute toxicity & Improve the overall survival rate	Allogenous	[180]
	-	Phase II	Transplant	Improve the overall survival rate	Allogenous	[181]
Type I diabetes	-	Phase I	Infusion	Preserve β-cell function	Autologous	[182]
Type II diabetes	-	Phase I	Infusion into the celiac and superior mesenteric arteries	Reduce HbA1C & fasting glucose	Autologous	[149]
	-	Phase I/II	Infusion into superior pancreaticoduodenal artery & splenic artery	Reduce insulin dosage	Autologous	[150]
		Phase I/II	Intravenously transfusion	Reduce HbA1C and insulin dosage	Autologous	[151]
	-	Phase I	Injection into superior pancreaticoduodenal	Reduce insulin dosage, improve insulin sensitivity	Autologous	[183]
Systemic lupus erythematosus	-	Phase I/II	Intravenous infusion	Induce overall survival rate & Achieve low disease activity (LDA) and clinical remission (CR)	Allogenous	[140,141]
	Preclinical	-	Infusion	Suppress Tfh cells development	Allogenous	[147]
Rheumatoid arthritis	Preclinical	-	Infusion	Reduce bone erosions, synovitis and articular destruction, TNF-α and IL-1β in serum & joints	Allogenous	[138]
	-	Phase I/II	Intra-articular knee implantation	Increase WOMAC, VAS, time to jelling and pain-free walking distance, standing time	Autologous	[139]
Multiple sclerosis	-	Phase I/II	Intrathecal injection	Improve Expanded Disability Scale Score (EDSS), vision & low contrast sensitivity	Autologous	[157]
Amyotrophic lateral sclerosis	-	Phase I/IIa	Intrathecal injection	Reduce ALSFRS	Autologous	[160]
	-	Phase I	Intravenous and intrathecal injection	Reduce ALS-FRS score and FVC percentage	Autologous	[161]
	-	Phase I	Intrathecal injection	Safe and feasible	Autologous	[162]
Parkinson’s disease	Preclinical	-	Intravenous injection	Improved dopamine transporter binding activity	Allogenous	[165]
Alzheimer disease	Preclinical	-	Intraventricularly injection	Improve behavior, brain damage & reduce cytokines	Allogenous	[167]
	Preclinical	-	Tail intravenous injection	Reduce inflammatory cytokines & regulate expression of Aβ-related genes	Allogenous	[166]
Osteoarthritis	-	Phase I/II	intra-articular infusion	Improve joint inflammation, OA cartilage organization	Autologous	[172]
Crohn’s Disease	-	Phase I/II	intrafistular injections	Rescue refractory patients & regain responsiveness to drugs	Autologous	[184]
	-	Phase I/II	Intrathecal injection	Promote healing of perianal fistulas	Allogenous	[185]
	-	Phase I	Intravenous infusion	Safe and feasible	Autologous	[186]
Cardiovascular diseases	-	Phase I/IIa	Surgical transplantation	Safe & Improve outcome of stroke	Allogenous	[187]
	-	Phase I/IIa	Transendocardial injection	Reduce SAE incidence & induce 6-min. walk test (treated allogenous BMSCs)	Allogenous vs. Autologous	[188]
	Preclinical	-	Local transplantation	Increase myocardium metabolism, glucose transporters & metabolism	Allogenous	[189]
Acute respiratory distress syndrome	-	Phase I	Intravenous infusion	None of these severe adverse events	Allogenous	[190]
Liver cirrhosis	-	Phase II	Injection	improve fibrosis quantification & liver function	Autologous	[191]
	Preclinical	-	Injection via portal or tail vein	Improve liver function& reduce ALT, serum hyaluronic acid, laminin and procollagen type III	Allogenous	[192]
Liver failure	-	Phase I/IIa	Intravenous infusion	Improve survival rate, liver function and decrease incidence of severe infections	Allogenous	[193]

## Abbreviations

BMSCs	Bone-marrow mesenchymal stem cells
UCSCs	Umbilical cord stem cells
ADSCs	Adipose-derived stem cells
ISCT	International Society for Cellular Therapy
MSCs	Mesenchymal stem cells
NK	Natural Killer
CFU-F	Clonogenic fibroblast precursor cells
GVHD	Graft versus host disease
EGFR	Epidermal growth factor receptor
IGFR	Insulin growth factor receptor
PDGFRα	Platelet-derived growth factor receptor alpha
GMP	Good manufacturing practices
ATMPs	Advanced therapy medicinal products
CGTP	Current Good Tissue Practice
FCS	Fetal calf serum
DMEM	Dulbecco Modified Eagle Medium
BMPs	Bone morphogenetic proteins
Runx2	Runt-related transcription 2
PPARγ2	Peroxisome proliferators-activated receptor gamma 2
SLE	Systemic lupus erythematosus
RH	Rheumatoid arthritis
HSCs	Hematopoietic stem cells
WOMAC	Western Ontario and McMaster Universities Arthritis Index
VAS	Visual analog scale
SLEDAI	SLE Disease Activity Index
ALS	Amyotrophic lateral sclerosis
PD	Parkinson’s disease
AD	Alzheimer disease
OA	Osteoarthritis

## References

[1] He Q., Ye Z., Zhou Y., Tan W.-S. (2018). Comparative study of mesenchymal stem cells from rat bone marrow and adipose tissue. Turk. J. Biol..

[2] Chan C.K.F., Gulati G.S., Sinha R., Tompkins J.V., Lopez M., Carter A.C., Ransom R.C., Reinisch A., Wearda T., Murphy M. (2018). Identification of the human skeletal stem cell. Cell.

[3] Yamada Y., Nakamura-Yamada S., Kusano K., Baba S. (2019). Clinical Potential and Current Progress of Dental Pulp Stem Cells for Various Systemic Diseases in Regenerative Medicine: A Concise Review. Int. J. Mol. Sci..

[4] Yang R., Liu F., Wang J., Chen X., Xie J., Xiong K. (2019). Epidermal stem cells in wound healing and their clinical applications. Stem Cell Res. Ther..

[5] Kelly P.N. (2017). Skin stem cells regenerate a human epidermis. Science.

[6] Wu M., Zhang R., Zou Q., Chen Y., Zhou M., Li X., Ran R., Chen Q. (2018). Comparison of the Biological Characteristics of Mesenchymal Stem Cells Derived from the Human Placenta and Umbilical Cord. Sci. Rep..

[7] Talwadekar M.D., Kale V.P., Limaye L.S. (2015). Placenta-derived mesenchymal stem cells possess better immunoregulatory properties compared to their cord-derived counterparts—A paired sample study. Sci. Rep..

[8] Dominici M., Le Blanc K., Mueller I., Slaper-Cortenbach I., Marini F.C., Krause D.S., Deans R.J., Keating A., Prockop D.J., Horwitz E.M. (2006). Minimal criteria for defining multipotent mesenchymal stromal cells. The International Society for Cellular Therapy position statement. Cytotherapy.

[9] Neri S. (2019). Genetic Stability of Mesenchymal Stromal Cells for Regenerative Medicine Applications: A Fundamental Biosafety Aspect. Int. J. Mol. Sci..

[10] Mazini L., Rochette L., Amine M., Malka G. (2019). Regenerative Capacity of Adipose Derived Stem Cells (ADSCs), Comparison with Mesenchymal Stem Cells (MSCs). Int. J. Mol. Sci..

[11] Wagner W., Wein F., Seckinger A., Frankhauser M., Wirkner U., Krause U., Blake J., Schwager C., Eckstein V., Ansorge W. (2005). Comparative characteristics of mesenchymal stem cells from human bone marrow, adipose tissue, and umbilical cord blood. Exp. Hematol..

[12] Musina R.A., Bekchanova E.S., Belyavskii A.V., Sukhikh G.T. (2006). Differentiation potential of mesenchymal stem cells of different origin. Bull. Exp. Biol. Med..

[13] Afanasyev B.V., Elstner E.E., Zander A.R. (2009). AJ Friedenstein, founder of the mesenchymal stem cell concept. Cell Ther. Transpl..

[14] Number of Studies Classified by Phase.

[15] Kramer J., Dazzi F., Dominici M., Schlenke P., Wagner W. (2012). Clinical perspectives of mesenchymal stem cells. Stem Cells Int..

[16] Jang Y., Koh Y.G., Choi Y.-J., Kim S.-H., Yoon D.S., Lee M., Lee J.W. (2015). Characterization of adipose tissue-derived stromal vascular fraction for clinical application to cartilage regeneration. In Vitro Cell. Dev. Biol. Anim..

[17] Kouroupis D., Sanjurjo-Rodriguez C., Jones E., Correa D. (2018). Mesenchymal Stem Cell Functionalization for Enhanced Therapeutic Applications. Tissue Eng. Part B.

[18] Yi T., Kim S.-N., Lee H.-J., Kim J., Cho Y.-K., Shin D.-H., Tak S.-J., Moon S.-H., Kang J.-E., Ji I.-M. (2015). Manufacture of Clinical-Grade Human Clonal Mesenchymal Stem Cell Products from Single Colony Forming Unit-Derived Colonies Based on the Subfractionation Culturing Method. Tissue Eng. Part C.

[19] Martin I., De Boer J., Sensebe L., The MSC Committee of the International Society for Cellular Therapy (2016). A relativity concept in mesenchymal stromal cell manufacturing. Cytotherapy.

[20] Zoremba M., Kalmus G., Steinfeldt T., Wulf H., Dette F. (2010). Respiratory impairment in the obese following general anesthesia–impact of anaesthesia and patient related factors. J. Anesthe Clin. Res..

[21] Lins Kusterer L.E.F. (2011). Oral Diseases and Liver Pre and Post-Transplantatio n Disorders. J. Transpl. Technol. Res. S.

[22] Jeevani T. (2011). Stem cell transplantation-types, risks and benefits. J. Stem Cell Res. Ther..

[23] Leventhal A., Chen G., Negro A., Boehm M. (2012). The benefits and risks of stem cell technology. Oral. Dis..

[24] Soldner F., Laganière J., Cheng A.W., Hockemeyer D., Gao Q., Alagappan R., Khurana V., Golbe L.I., Myers R.H., Lindquist S. (2011). Generation of isogenic pluripotent stem cells differing exclusively at two early onset Parkinson point mutations. Cell.

[25] Liu G.-H., Suzuki K., Qu J., Sancho-Martinez I., Yi F., Li M., Kumar S., Nivet E., Kim J., Soligalla R.D. (2011). Targeted gene correction of laminopathy-associated LMNA mutations in patient-specific iPSCs. Cell Stem Cell.

[26] Champlin R., Kufe D.W., Pollock R.E., Weichselbaum R.R. (2003). Selection of autologous or allogeneic transplantation. Holland-Frei Cancer Medicine.

[27] Map of Clinical Trials on Bone Marrow Stem Cells in the World. http://www.clinicals.gov.

[28] Friedenstein A.J., Gorskaja J.F., Kulagina N.N. (1976). Fibroblast precursors in normal and irradiated mouse hematopoietic organs. Exp. Hematol..

[29] Samsonraj R.M., Raghunath M., Nurcombe V., Hui J.H., van Wijnen A.J., Cool S.M. (2017). Concise Review: Multifaceted Characterization of Human Mesenchymal Stem Cells for Use in Regenerative Medicine. Stem Cells Transl. Med..

[30] Nasef A., Fouillard L., El-Taguri A., Lopez M. (2007). Human bone marrow-derived mesenchymal stem cells. Libyan J. Med..

[31] Andrzejewska A., Lukomska B., Janowski M. (2019). Concise Review: Mesenchymal Stem Cells: From Roots to Boost. Concise Rev..

[32] Cakouros D., Gronthos S. (2019). Epigenetic Regulation of Bone Marrow Stem Cell Aging: Revealing Epigenetic Signatures associated with Hematopoietic and Mesenchymal Stem Cell Aging. Aging Dis..

[33] Beane O.S., Fonseca V.C., Cooper L.L., Koren G., Darling E.M. (2014). Impact of aging on the regenerative properties of bone marrow-, muscle-, and adipose-derived mesenchymal stem/stromal cells. PLoS ONE.

[34] Shen J., Tsai Y.-T., Dimarco N.M., Long M.A., Sun X., Tang L. (2011). Transplantation of mesenchymal stem cells from young donors delays aging in mice. Sci. Rep..

[35] Stolzing A., Jones E., McGonagle D., Scutt A. (2008). Age-related changes in human bone marrow-derived mesenchymal stem cells: Consequences for cell therapies. Mech. Ageing Dev..

[36] Hillyer C.D., Josephson C.D., Blajchman M.A., Vostal J.G., Epstein J.S., Goodman J.L. (2003). Bacterial contamination of blood components: Risks, strategies, and regulation: Joint ASH and AABB educational session in transfusion medicine. ASH Educ. Program Book.

[37] Ljungman P., Lawler M., ÅSjo B., Bogdanovic G., Karlsson K., Malm C., McCann S.R., Ringdén O., Gahrton G. (1994). Infection of donor lymphocytes with human T lymphotrophic virus type 1 (HTLV-I) following allogeneic bone marrow transplantation for HTLV-I positive adult T-cell leukaemia. Br. J. Haematol..

[38] Gratwohl A., Corny P., Speck B. (1979). Bone marrow transplantation from a donor with Gaucher’s disease. Transplantation.

[39] Mushahary D., Spittler A., Kasper C., Weber V., Charwat V. (2018). Isolation, cultivation, and characterization of human mesenchymal stem cells. Cytom. Part A.

[40] Suliman S., Ali H.R.W., Karlsen T.A., Amiaud J., Mohamed-Ahmed S., Layrolle P., Costea D.E., Brinchmann J.E., Mustafa K. (2019). Impact of humanised isolation and culture conditions on stemness and osteogenic potential of bone marrow derived mesenchymal stromal cells. Sci. Rep..

[41] Amorin B., Alegretti A.P., Valim V., Pezzi A., Laureano A.M., da Silva M.A.L., Wieck A., Silla L. (2014). Mesenchymal stem cell therapy and acute graft-versus-host disease: A review. Hum. Cell.

[42] Mattiucci D., Maurizi G., Leoni P., Poloni A. (2018). Aging-and Senescence-associated Changes of Mesenchymal Stromal Cells in Myelodysplastic Syndromes. Cell Transplant..

[43] De Miguel M., Fuentes-Julian S., Blazquez-Martinez A., Pascual C., Aller M., Arias J., Arnalich-Montiel F. (2012). Immunosuppressive properties of mesenchymal stem cells: Advances and applications. Curr. Mol. Med..

[44] Abdal Dayem A., Lee S.B., Kim K., Lim K.M., Jeon T.-I., Seok J. (2019). Production of Mesenchymal Stem Cells through Stem Cell Reprogramming. Int. J. Mol. Sci..

[45] Wu L., Leijten J., van Blitterswijk C.A., Karperien M. (2013). Fibroblast growth factor-1 is a mesenchymal stromal cell-secreted factor stimulating proliferation of osteoarthritic chondrocytes in co-culture. Stem Cells Dev..

[46] Kinnaird T., Stabile E., Burnett M.S., Lee C.W., Barr S., Fuchs S., Epstein S.E. (2004). Marrow-derived stromal cells express genes encoding a broad spectrum of arteriogenic cytokines and promote in vitro and in vivo arteriogenesis through paracrine mechanisms. Circ. Res..

[47] Imberti B., Morigi M., Tomasoni S., Rota C., Corna D., Longaretti L., Rottoli D., Valsecchi F., Benigni A., Wang J. (2007). Insulin-like growth factor-1 sustains stem cell–mediated renal repair. J. Am. Soc. Nephrol..

[48] Dufourcq P., Descamps B., Tojais N.F., Leroux L., Oses P., Daret D., Moreau C., Lamazière J.M.D., Couffinhal T., Duplaa C. (2008). Secreted frizzled-related protein-1 enhances mesenchymal stem cell function in angiogenesis and contributes to neovessel maturation. Stem Cells.

[49] Mirotsou M., Zhang Z., Deb A., Zhang L., Gnecchi M., Noiseux N., Mu H., Pachori A., Dzau V. (2007). Secreted frizzled related protein 2 (Sfrp2) is the key Akt-mesenchymal stem cell-released paracrine factor mediating myocardial survival and repair. Proc. Natl. Acad. Sci. USA.

[50] Block G.J., Ohkouchi S., Fung F., Frenkel J., Gregory C., Pochampally R., DiMattia G., Sullivan D.E., Prockop D.J. (2009). Multipotent stromal cells are activated to reduce apoptosis in part by upregulation and secretion of stanniocalcin-1. Stem Cells.

[51] Baglio S.R., Rooijers K., Koppers-Lalic D., Verweij F.J., Lanzón M.P., Zini N., Naaijkens B., Perut F., Niessen H.W.M., Baldini N. (2015). Human bone marrow-and adipose-mesenchymal stem cells secrete exosomes enriched in distinctive miRNA and tRNA species. Stem Cell Res. Ther..

[52] Kim Y.J., Kim H.K., Cho H.H., Bae Y.C., Suh K.T., Jung J.S. (2007). Direct comparison of human mesenchymal stem cells derived from adipose tissues and bone marrow in mediating neovascularization in response to vascular ischemia. Cell. Physiol. Biochem..

[53] Sun L., Cui M., Wang Z., Feng X., Mao J., Chen P., Kangtao M., Chen F., Zhou C. (2007). Mesenchymal stem cells modified with angiopoietin-1 improve remodeling in a rat model of acute myocardial infarction. Biochem. Biophys. Res. Commun..

[54] Chen L., Xu Y., Zhao J., Zhang Z., Yang R., Xie J., Liu X., Qi S. (2014). Conditioned medium from hypoxic bone marrow-derived mesenchymal stem cells enhances wound healing in mice. PLoS ONE.

[55] Ma T., Chen Y., Chen Y., Meng Q., Sun J., Shao L., Yu Y., Huang H., Hu Y., Yang Z. (2018). MicroRNA-132, delivered by mesenchymal stem cell-derived exosomes, promote angiogenesis in myocardial infarction. Stem Cells Int..

[56] Di Nicola M., Carlo-Stella C., Magni M., Milanesi M., Longoni P.D., Matteucci P., Grisanti S., Gianni A.M. (2002). Human bone marrow stromal cells suppress T-lymphocyte proliferation induced by cellular or nonspecific mitogenic stimuli. Blood.

[57] Selmani Z., Naji A., Zidi I., Favier B., Gaiffe E., Obert L., Borg C., Saas P., Tiberghien P., Rouas-Freiss N. (2008). Human leukocyte antigen-G5 secretion by human mesenchymal stem cells is required to suppress T lymphocyte and natural killer function and to induce CD4 + CD25highFOXP3 + regulatory T cells. Stem Cells.

[58] Krampera M., Cosmi L., Angeli R., Pasini A., Liotta F., Andreini A., Santarlasci V., Mazzinghi B., Pizzolo G., Vinante F. (2006). Role for interferon-γ in the immunomodulatory activity of human bone marrow mesenchymal stem cells. Stem Cells.

[59] Meisel R., Zibert A., Laryea M., Göbel U., Däubener W., Dilloo D. (2004). Human bone marrow stromal cells inhibit allogeneic T-cell responses by indoleamine 2, 3-dioxygenase–mediated tryptophan degradation. Blood.

[60] Sato K., Ozaki K., Oh I., Meguro A., Hatanaka K., Nagai T., Muroi K., Ozawa K. (2006). Nitric oxide plays a critical role in suppression of T-cell proliferation by mesenchymal stem cells. Blood.

[61] Haynesworth S.E., Baber M.A., Caplan A.I. (1996). Cytokine expression by human marrow-derived mesenchymal progenitor cells in vitro: Effects of dexamethasone and IL-1α. J. Cell. Physiol..

[62] Gupta N., Su X., Popov B., Lee J.W., Serikov V., Matthay M.A. (2007). Intrapulmonary delivery of bone marrow-derived mesenchymal stem cells improves survival and attenuates endotoxin-induced acute lung injury in mice. J. Immunol..

[63] Nasef A., Mazurier C., Bouchet S., François S., Chapel A., Thierry D., Gorin N.-C., Fouillard L. (2008). Leukemia inhibitory factor: Role in human mesenchymal stem cells mediated immunosuppression. Cell. Immunol..

[64] Németh K., Leelahavanichkul A., Yuen P.S.T., Mayer B., Parmelee A., Doi K., Robey P.G., Leelahavanichkul K., Koller B.H., Brown J.M. (2009). Bone marrow stromal cells attenuate sepsis via prostaglandin E 2–dependent reprogramming of host macrophages to increase their interleukin-10 production. Nat. Med..

[65] Rice C.M., Marks D.I., Walsh P., Kane N.M., Guttridge M.G., Redondo J., Sarkar P., Owen D., Wilkins A., Scolding N.J. (2015). Repeat infusion of autologous bone marrow cells in multiple sclerosis: Protocol for a phase I extension study (SIAMMS-II). BMJ Open.

[66] Sürder D., Manka R., Moccetti T., Lo Cicero V., Emmert Maximilian Y., Klersy C., Soncin S., Turchetto L., Radrizzani M., Zuber M. (2016). Effect of Bone Marrow–Derived Mononuclear Cell Treatment, Early or Late After Acute Myocardial Infarction. Circ. Res..

[67] Al Demour S., Jafar H., Adwan S., AlSharif A., Alhawari H., Alrabadi A., Zayed A., Jaradat A., Awidi A. (2018). Safety and Potential Therapeutic Effect of Two Intracavernous Autologous Bone Marrow Derived Mesenchymal Stem Cells injections in Diabetic Patients with Erectile Dysfunction: An Open Label Phase I Clinical Trial. Urol. Int..

[68] Qi Z., Liu S., Duan F. (2018). Effects of bone marrow mononuclear cells delivered through a graft vessel in patients with previous myocardial infarction and chronic heart failure: An echocardiographic study of left ventricular dyssynchrony. J. Clin. Ultrasound.

[69] Huang S., Xu L., Sun Y., Wu T., Wang K., Li G. (2015). An improved protocol for isolation and culture of mesenchymal stem cells from mouse bone marrow. J. Orthop. Transl..

[70] Zhu H., Guo Z.-K., Jiang X.-X., Li H., Wang X.-Y., Yao H.-Y., Zhang Y., Mao N. (2010). A protocol for isolation and culture of mesenchymal stem cells from mouse compact bone. Nat. Protoc..

[71] Yusop N., Battersby P., Alraies A., Sloan A.J., Moseley R., Waddington R.J. (2018). Isolation and Characterisation of Mesenchymal Stem Cells from Rat Bone Marrow and the Endosteal Niche: A Comparative Study. Stem Cells Int..

[72] Mabuchi Y., Houlihan D.D., Akazawa C., Okano H., Matsuzaki Y. (2013). Prospective isolation of murine and human bone marrow mesenchymal stem cells based on surface markers. Stem Cells Int..

[73] Ghazanfari R., Zacharaki D., Li H., Ching Lim H., Soneji S., Scheding S. (2017). Human Primary Bone Marrow Mesenchymal Stromal Cells and Their in vitro Progenies Display Distinct Transcriptional Profile Signatures. Sci. Rep..

[74] McDaniel J.S., Antebi B., Pilia M., Hurtgen B.J., Belenkiy S., Necsoiu C., Cancio L.C., Rathbone C.R., Batchinsky A.I. (2017). Quantitative assessment of optimal bone marrow site for the isolation of porcine mesenchymal stem cells. Stem Cells Int..

[75] Baghaei K., Hashemi S.M., Tokhanbigli S., Asadi Rad A., Assadzadeh-Aghdaei H., Sharifian A., Zali M.R. (2017). Isolation, differentiation, and characterization of mesenchymal stem cells from human bone marrow. Gastroenterol. Hepatol. Bed Bench.

[76] Li H., Ghazanfari R., Zacharaki D., Lim H.C., Scheding S. (2016). Isolation and characterization of primary bone marrow mesenchymal stromal cells. Ann. N. Y. Acad. Sci..

[77] Lee J.-S., Kim S.-K., Cha J.-K., Jung B.-J., Choi S.-B., Choi E.-Y., Kim C.-S. (2016). Novel Technique for Isolating Human Bone Marrow Stem Cells Using Hyaluronic Acid Hydrogel. Tissue Eng. Part C.

[78] Miura Y. (2016). Human bone marrow mesenchymal stromal/stem cells: Current clinical applications and potential for hematology. Int. J. Hematol..

[79] Gang E.J., Bosnakovski D., Figueiredo C.A., Visser J.W., Perlingeiro R.C.R. (2007). SSEA-4 identifies mesenchymal stem cells from bone marrow. Blood.

[80] Lv F.-J., Tuan R.S., Cheung K.M.C., Leung V.Y.L. (2014). Concise Review: The Surface Markers and Identity of Human Mesenchymal Stem Cells. Stem Cells.

[81] Fekete N., Rojewski M.T., Fürst D., Kreja L., Ignatius A., Dausend J., Schrezenmeier H. (2012). GMP-Compliant Isolation and Large-Scale Expansion of Bone Marrow-Derived MSC. PLoS ONE.

[82] Barckhausen C., Rice B., Baila S., Sensebé L., Schrezenmeier H., Nold P., Hackstein H., Rojewski M.T. (2016). GMP-compliant expansion of clinical-grade human mesenchymal stromal/stem cells using a closed hollow fiber bioreactor. Mesenchymal Stem Cells.

[83] Rojewski M.T., Fekete N., Baila S., Nguyen K., Fürst D., Antwiler D., Dausend J., Kreja L., Ignatius A., Sensebé L. (2013). GMP-compliant isolation and expansion of bone marrow-derived MSCs in the closed, automated device quantum cell expansion system. Cell Transplant..

[84] Subbanna P.K. (2007). Mesenchymal stem cells for treating GVHD: In-vivo fate and optimal dose. Med. Hypotheses.

[85] Aijaz A., Li M., Smith D., Khong D., LeBlon C., Fenton O.S., Olabisi R.M., Libutti S., Tischfield J., Maus M.V. (2018). Biomanufacturing for clinically advanced cell therapies. Nat. Biomed. Eng..

[86] European P., The Council of the European (2007). Regulation (EC) No 1394/2007 of the European Parliament and of the Council of 13 November 2007 on advanced therapy medicinal products and amending Directive 2001/83/EC and Regulation (EC) No 726/2004. Off. J..

[87] Sensebé L., Krampera M., Schrezenmeier H., Bourin P., Giordano R. (2010). Mesenchymal stem cells for clinical application. Vox Sang..

[88] Korrapati N., Nanganuru H.Y. (2014). Perfusion Method for Intra-bone Marrow Collection and Stem Cell Transplantation: A Critical Review. Curr. Stem Cell Res. Ther..

[89] Colter D.C., Sekiya I., Prockop D.J. (2001). Identification of a subpopulation of rapidly self-renewing and multipotential adult stem cells in colonies of human marrow stromal cells. Proc. Natl. Acad. Sci. USA.

[90] Van Pham P., Vu N.B. (2016). In vitro expansion of mesenchymal stem cells for clinical use. Prog. Stem Cell.

[91] Hagmann S., Moradi B., Frank S., Dreher T., Kämmerer P.W., Richter W., Gotterbarm T. (2013). FGF-2 addition during expansion of human bone marrow-derived stromal cells alters MSC surface marker distribution and chondrogenic differentiation potential. Cell Prolif..

[92] Chase L.G., Yang S., Zachar V., Yang Z., Lakshmipathy U., Bradford J., Boucher S.E., Vemuri M.C. (2012). Development and characterization of a clinically compliant xeno-free culture medium in good manufacturing practice for human multipotent mesenchymal stem cells. Stem Cells Transl. Med..

[93] Bieback K., Schallmoser K., Klüter H., Strunk D. (2008). Clinical Protocols for the Isolation and Expansion of Mesenchymal Stromal Cells. Transfus. Med. Hemother..

[94] Russell A.L., Lefavor R.C., Zubair A.C. (2018). Characterization and cost–benefit analysis of automated bioreactor-expanded mesenchymal stem cells for clinical applications. Transfusion.

[95] Yang Y.H.K., Ogando C.R., See C.W., Chang T.-Y., Barabino G.A. (2018). Changes in phenotype and differentiation potential of human mesenchymal stem cells aging in vitro. Stem Cell Res. Ther..

[96] Bernardo M.E., Zaffaroni N., Novara F., Cometa A.M., Avanzini M.A., Moretta A., Montagna D., Maccario R., Villa R., Daidone M.G. (2007). Human bone marrow–derived mesenchymal stem cells do not undergo transformation after long-term in vitro culture and do not exhibit telomere maintenance mechanisms. Cancer Res..

[97] Kretlow J.D., Jin Y.-Q., Liu W., Zhang W.J., Hong T.-H., Zhou G., Baggett L.S., Mikos A.G., Cao Y. (2008). Donor age and cell passage affects differentiation potential of murine bone marrow-derived stem cells. BMC Cell Biol..

[98] Zheng Y.-H., Xiong W., Su K., Kuang S.-J., Zhang Z.-G. (2013). Multilineage differentiation of human bone marrow mesenchymal stem cells in vitro and in vivo. Exp. Ther. Med..

[99] Wang C., Meng H., Wang X., Zhao C., Peng J., Wang Y. (2016). Differentiation of Bone Marrow Mesenchymal Stem Cells in Osteoblasts and Adipocytes and its Role in Treatment of Osteoporosis. Med. Sci. Monit..

[100] Okolicsanyi R.K., Camilleri E.T., Oikari L.E., Yu C., Cool S.M., Van Wijnen A.J., Griffiths L.R., Haupt L.M. (2015). Human mesenchymal stem cells retain multilineage differentiation capacity including neural marker expression after extended in vitro expansion. PLoS ONE.

[101] Moghadam F.H., Tayebi T., Dehghan M., Eslami G., Nadri H., Moradi A., Vahedian-Ardakani H., Barzegar K. (2014). Differentiation of bone marrow mesenchymal stem cells into chondrocytes after short term culture in alkaline medium. Int. J. Hematol. Oncol. Stem Cell Res..

[102] Glynn E.R.A., Londono A.S., Zinn S.A., Hoagland T.A., Govoni K.E. (2013). Culture conditions for equine bone marrow mesenchymal stem cells and expression of key transcription factors during their differentiation into osteoblasts. J. Anim. Sci. Biotechnol..

[103] Banerjee C., Javed A., Choi J.-Y., Green J., Rosen V., Van Wijnen A.J., Stein J.L., Lian J.B., Stein G.S. (2001). Differential regulation of the two principal Runx2/Cbfa1 n-terminal isoforms in response to bone morphogenetic protein-2 during development of the osteoblast phenotype. Endocrinology.

[104] Bae Y.-J., Kwon Y.-R., Kim H.J., Lee S., Kim Y.-J. (2017). Enhanced differentiation of mesenchymal stromal cells by three-dimensional culture and azacitidine. Blood Res..

[105] Cunha C.A., Lira F.S., Rosa Neto J.C., Pimentel G.D., Souza G.I., da Silva C.M.G., de Souza C.T., Ribeiro E.B., Sawaya A.C.H.F., Oller do Nascimento C.M. (2013). Green tea extract supplementation induces the lipolytic pathway, attenuates obesity, and reduces low-grade inflammation in mice fed a high-fat diet. Mediat. Inflamm..

[106] Chen X., Zhi X., Wang J., Su J. (2018). RANKL signaling in bone marrow mesenchymal stem cells negatively regulates osteoblastic bone formation. Bone Res..

[107] Wang J., Liu S., Li J., Zhao S., Yi Z. (2019). Roles for miRNAs in osteogenic differentiation of bone marrow mesenchymal stem cells. Stem Cell Res. Ther..

[108] Chen Y., Yang Y.-R., Fan X.-L., Lin P., Yang H., Chen X.-Z., Xu X.-D. (2019). miR-206 inhibits osteogenic differentiation of bone marrow mesenchymal stem cells by targetting glutaminase. Biosci. Rep..

[109] Wu J., Zhang W., Ran Q., Xiang Y., Zhong J.F., Li S.C., Li Z. (2018). The differentiation balance of bone marrow mesenchymal stem cells is crucial to hematopoiesis. Stem Cells Int..

[110] Song L.I.N., Tuan R.S. (2004). Transdifferentiation potential of human mesenchymal stem cells derived from bone marrow. FASEB J..

[111] Yannarelli G., Pacienza N., Montanari S., Santa-Cruz D., Viswanathan S., Keating A. (2017). OCT4 expression mediates partial cardiomyocyte reprogramming of mesenchymal stromal cells. PLoS ONE.

[112] Liu Y., Jiang X., Zhang X., Chen R., Sun T., Fok K.L., Dong J., Tsang L.L., Yi S., Ruan Y. (2011). Dedifferentiation-reprogrammed mesenchymal stem cells with improved therapeutic potential. Stem Cells.

[113] Sohn H.S., Heo J.S., Kim H.-S., Choi Y., Kim H.O. (2013). Duration of in vitro storage affects the key stem cell features of human bone marrow-derived mesenchymal stromal cells for clinical transplantation. Cytotherapy.

[114] Harris D.T. (2016). Long-term frozen storage of stem cells: Challenges and solutions. J. Biorepos. Sci. Appl. Med..

[115] Petrenko Y., Chudickova M., Vackova I., Groh T., Kosnarova E., Cejkova J., Turnovcova K., Petrenko A., Sykova E., Kubinova S. (2019). Clinically Relevant Solution for the Hypothermic Storage and Transportation of Human Multipotent Mesenchymal Stromal Cells. Stem Cells Int..

[116] Aoyama T. (2017). Transportation of Mesenchymal Stem Cells for Clinical Applications. Mesenchymal Stem Cells.

[117] Harris D.T. (2014). Stem Cell Banking for Regenerative and Personalized Medicine. Biomedicines.

[118] Bloom D.D., Centanni J.M., Bhatia N., Emler C.A., Drier D., Leverson G.E., McKenna D.H., Gee A.P., Lindblad R., Hei D.J. (2015). A reproducible immunopotency assay to measure mesenchymal stromal cell–mediated T-cell suppression. Cytotherapy.

[119] Salem B., Miner S., Hensel N.F., Battiwalla M., Keyvanfar K., Stroncek D.F., Gee A.P., Hanley P.J., Bollard C.M., Ito S. (2015). Quantitative activation suppression assay to evaluate human bone marrow–derived mesenchymal stromal cell potency. Cytotherapy.

[120] Lee S., Lai W., Yu S., Chen H., Shen P., Lin S. (2019). Developing a flow cytometry-based quantitative ido assay to measure immune potency of mesenchymal stromal cells product for phase i clinical trial. Cytotherapy.

[121] Chinnadurai R., Rajan D., Qayed M., Arafat D., Garcia M., Liu Y., Kugathasan S., Anderson L.J., Gibson G., Galipeau J. (2018). Potency Analysis of Mesenchymal Stromal Cells Using a Combinatorial Assay Matrix Approach. Cell Rep..

[122] Shojaei F., Rahmati S., Banitalebi Dehkordi M. (2019). A review on different methods to increase the efficiency of mesenchymal stem cell-based wound therapy. Wound Repair Regen..

[123] Gonzalez M.E., Martin E.E., Anwar T., Arellano-Garcia C., Medhora N., Lama A., Chen Y.-C., Tanager K.S., Yoon E., Kidwell K.M. (2017). Mesenchymal stem cell-induced DDR2 mediates stromal-breast cancer interactions and metastasis growth. Cell Rep..

[124] Huang J.C., Basu S.K., Zhao X., Chien S., Fang M., Oehler V.G., Appelbaum F.R., Becker P.S. (2015). Mesenchymal stromal cells derived from acute myeloid leukemia bone marrow exhibit aberrant cytogenetics and cytokine elaboration. Blood Cancer J..

[125] He L., Zhao F., Zheng Y., Wan Y., Song J. (2016). Loss of interactions between p53 and survivin gene in mesenchymal stem cells after spontaneous transformation in vitro. Int. J. Biochem. Cell Biol..

[126] Pelizzo G., Avanzini M.A., Folini M., Bussani R., Mantelli M., Croce S., Acquafredda G., Travaglino P., Cimino-Reale G., Boni M. (2017). CPAM type 2-derived mesenchymal stem cells: Malignancy risk study in a 14-month-old boy. Pediatric Pulmonol..

[127] Djouad F., Plence P., Bony C., Tropel P., Apparailly F., Sany J., Noël D., Jorgensen C. (2003). Immunosuppressive effect of mesenchymal stem cells favors tumor growth in allogeneic animals. Blood.

[128] Karnoub A.E., Dash A.B., Vo A.P., Sullivan A., Brooks M.W., Bell G.W., Richardson A.L., Polyak K., Tubo R., Weinberg R.A. (2007). Mesenchymal stem cells within tumour stroma promote breast cancer metastasis. Nature.

[129] Tolar J., Nauta A.J., Osborn M.J., Panoskaltsis Mortari A., McElmurry R.T., Bell S., Xia L., Zhou N., Riddle M., Schroeder T.M. (2007). Sarcoma derived from cultured mesenchymal stem cells. Stem Cells.

[130] Lee S., Choi E., Cha M.-J., Hwang K.-C. (2015). Cell adhesion and long-term survival of transplanted mesenchymal stem cells: A prerequisite for cell therapy. Oxidative Med. Cell. Longev..

[131] Wang M., Yuan Q., Xie L. (2018). Mesenchymal Stem Cell-Based Immunomodulation: Properties and Clinical Application. Stem Cells Int..

[132] Zhou H., Guo M., Bian C., Sun Z., Yang Z., Zeng Y., Ai H., Zhao R.C. (2010). Efficacy of bone marrow-derived mesenchymal stem cells in the treatment of sclerodermatous chronic graft-versus-host disease: Clinical report. Biol. Blood Marrow Transplant..

[133] Prasad V.K., Lucas K.G., Kleiner G.I., Talano J.A.M., Jacobsohn D., Broadwater G., Monroy R., Kurtzberg J. (2011). Efficacy and safety of ex vivo cultured adult human mesenchymal stem cells (Prochymal™) in pediatric patients with severe refractory acute graft-versus-host disease in a compassionate use study. Biol. Blood Marrow Transplant..

[134] Kebriaei P., Isola L., Bahceci E., Holland K., Rowley S., McGuirk J., Devetten M., Jansen J., Herzig R., Schuster M. (2009). Adult human mesenchymal stem cells added to corticosteroid therapy for the treatment of acute graft-versus-host disease. Biol. Blood Marrow Transplant..

[135] Zhao L., Chen S., Yang P., Cao H., Li L. (2019). The role of mesenchymal stem cells in hematopoietic stem cell transplantation: Prevention and treatment of graft-versus-host disease. Stem Cell Res. Ther..

[136] Farini A., Sitzia C., Erratico S., Meregalli M., Torrente Y. (2014). Clinical applications of mesenchymal stem cells in chronic diseases. Stem Cells Int..

[137] Bosi C.A., Lanzoni G., Pugliese A. (2016). Clinical trials of mesenchymal stem cell transplantation in patients with type 1 diabetes and systemic lupus erythematosus: Is it time for larger studies. CellR4.

[138] Zhang Q., Li Q., Zhu J., Guo H., Zhai Q., Li B., Jin Y., He X., Jin F. (2019). Comparison of therapeutic effects of different mesenchymal stem cells on rheumatoid arthritis in mice. Peer J..

[139] Shadmanfar S., Labibzadeh N., Emadedin M., Jaroughi N., Azimian V., Mardpour S., Kakroodi F.A., Bolurieh T., Hosseini S.E., Chehrazi M. (2018). Intra-articular knee implantation of autologous bone marrow–derived mesenchymal stromal cells in rheumatoid arthritis patients with knee involvement: Results of a randomized, triple-blind, placebo-controlled phase 1/2 clinical trial. Cytotherapy.

[140] Wang D., Zhang H., Liang J., Wang H., Hua B., Feng X., Gilkeson G.S., Farge D., Shi S., Sun L. (2018). A long-term follow-up study of allogeneic mesenchymal stem/stromal cell transplantation in patients with drug-resistant systemic lupus erythematosus. Stem Cell Rep..

[141] Wen L., Labopin M., Badoglio M., Wang D., Sun L., Farge-Bancel D. (2019). Prognostic Factors for Clinical Response in Systemic Lupus Erythematosus Patients Treated by Allogeneic Mesenchymal Stem Cells. Stem Cells Int..

[142] Sharma J., Hampton J.M., Valiente G.R., Wada T., Steigelman H., Young M.C., Spurbeck R.R., Blazek A.D., Bösh S., Jarjour W.N. (2017). Therapeutic development of mesenchymal stem cells or their extracellular vesicles to inhibit autoimmune-mediated inflammatory processes in systemic lupus erythematosus. Front. Immunol..

[143] Ji J., Wu Y., Meng Y., Zhang L., Feng G., Xia Y., Xue W., Zhao S., Gu Z., Shao X. (2017). JAK-STAT signaling mediates the senescence of bone marrow-mesenchymal stem cells from systemic lupus erythematosus patients. Acta Biochim. Biophys. Sin..

[144] Tan W., Gu Z., Shen B., Jiang J., Meng Y., Da Z., Liu H., Tao T., Cheng C. (2015). PTEN/Akt-p27kip1 signaling promote the BM-MSCs senescence and apoptosis in SLE patients. J. Cell. Biochem..

[145] Gu Z., Jiang J., Tan W., Xia Y., Cao H., Meng Y., Da Z., Liu H., Cheng C. (2013). p53/p21 Pathway involved in mediating cellular senescence of bone marrow-derived mesenchymal stem cells from systemic lupus erythematosus patients. Clin. Dev. Immunol..

[146] Gu Z., Tan W., Feng G., Meng Y., Shen B., Liu H., Cheng C. (2014). Wnt/β-catenin signaling mediates the senescence of bone marrow-mesenchymal stem cells from systemic lupus erythematosus patients through the p53/p21 pathway. Mol. Cell. Biochem..

[147] Jang E., Jeong M., Kim S., Jang K., Kang B.-K., Lee D.Y., Bae S.-C., Kim K.S., Youn J. (2016). Infusion of human bone marrow-derived mesenchymal stem cells alleviates autoimmune nephritis in a lupus model by suppressing follicular helper T-cell development. Cell Transplant..

[148] Li M., Ikehara S. (2013). Bone marrow stem cell as a potential treatment for diabetes. J. Diabetes Res..

[149] Wehbe T., Chahine N.A., Sissi S., Abou-Joaude I., Chalhoub L. (2016). Bone marrow derived stem cell therapy for type 2 diabetes mellitus. Stem Cell Investig..

[150] Sood V., Bhansali A., Mittal B.R., Singh B., Marwaha N., Jain A., Khandelwal N. (2017). Autologous bone marrow derived stem cell therapy in patients with type 2 diabetes mellitus-defining adequate administration methods. World J. Diabetes.

[151] Le P.T.-B., Phu-Van Doan N., Van Tien P., Hoang D.N.C., Phan N.K., Van Pham P. (2019). A type 2 diabetes mellitus patient was successfully treated by autologous bone marrow-derived stem cell transplantation: A case report. Biomed. Res. Ther..

[152] Phinney D.G., Pittenger M.F. (2017). Concise Review: MSC-Derived Exosomes for Cell-Free Therapy. Stem Cells.

[153] Qiu G., Zheng G., Ge M., Wang J., Huang R., Shu Q., Xu J. (2018). Mesenchymal stem cell-derived extracellular vesicles affect disease outcomes via transfer of microRNAs. Stem Cell Res. Ther..

[154] Akyurekli C., Le Y., Richardson R.B., Fergusson D., Tay J., Allan D.S. (2015). A systematic review of preclinical studies on the therapeutic potential of mesenchymal stromal cell-derived microvesicles. Stem Cell Rev. Rep..

[155] Elahi F.M., Farwell D.G., Nolta J.A., Anderson J.D. (2019). Concise Review: Preclinical Translation of Exosomes Derived from Mesenchymal Stem/Stromal Cells. Stem Cells.

[156] Baek G., Choi H., Kim Y., Lee H.-C., Choi C. (2019). Mesenchymal Stem Cell-Derived Extracellular Vesicles as Therapeutics and as a Drug Delivery Platform. Stem Cells Transl. Med..

[157] Yamout B., Hourani R., Salti H., Barada W., El-Hajj T., Al-Kutoubi A., Herlopian A., Baz E.K., Mahfouz R., Khalil-Hamdan R. (2010). Bone marrow mesenchymal stem cell transplantation in patients with multiple sclerosis: A pilot study. J. Neuroimmunol..

[158] Martino G., Franklin R.J.M., Van Evercooren A.B., Kerr D.A., Stem Cells in Multiple Sclerosis Consensus G. (2010). Stem cell transplantation in multiple sclerosis: Current status and future prospects. Nat. Rev. Neurol..

[159] Uccelli A., Laroni A., Brundin L., Clanet M., Fernandez O., Nabavi S.M., Muraro P.A., Oliveri R.S., Radue E.W., Sellner J. (2019). MEsenchymal StEm cells for Multiple Sclerosis (MESEMS): A randomized, double blind, cross-over phase I/II clinical trial with autologous mesenchymal stem cells for the therapy of multiple sclerosis. Trials.

[160] Syková E., Rychmach P., Drahorádová I., Konrádová Š., Růžičková K., Voříšek I., Forostyak S., Homola A., Bojar M. (2017). Transplantation of mesenchymal stromal cells in patients with amyotrophic lateral sclerosis: Results of phase I/IIa clinical trial. Cell Transplant..

[161] Nabavi S.M., Arab L., Jarooghi N., Bolurieh T., Abbasi F., Mardpour S., Azimyian V., Moeininia F., Maroufizadeh S., Sanjari L. (2018). Safety, feasibility of intravenous and intrathecal injection of autologous bone marrow derived mesenchymal stromal cells in patients with amyotrophic lateral sclerosis: An open label phase I clinical trial. Cell J..

[162] Oh K.-W., Moon C., Kim H.Y., Oh S.-I., Park J., Lee J.H., Chang I.Y., Kim K.S., Kim S.H. (2015). Phase I trial of repeated intrathecal autologous bone marrow-derived mesenchymal stromal cells in amyotrophic lateral sclerosis. Stem Cells Transl. Med..

[163] Goodarzi P., Aghayan H.R., Larijani B., Soleimani M., Dehpour A.-R., Sahebjam M., Ghaderi F., Arjmand B. (2015). Stem cell-based approach for the treatment of Parkinson’s disease. Med. J. Islam. Repub. Iran.

[164] Kitada M., Dezawa M. (2012). Parkinson’s disease and mesenchymal stem cells: Potential for cell-based therapy. Parkinson Dis..

[165] Park B.-N., Kim J.-H., Lee K., Park S.H., An Y.-S. (2015). Improved dopamine transporter binding activity after bone marrow mesenchymal stem cell transplantation in a rat model of Parkinson’s disease: Small animal positron emission tomography study with F-18 FP-CIT. Eur. Radiol..

[166] Wei Y., Xie Z., Bi J., Zhu Z. (2018). Anti-inflammatory effects of bone marrow mesenchymal stem cells on mice with Alzheimer’s disease. Exp. Ther. Med..

[167] Huang P., Freeman W.D., Edenfield B.H., Brott T.G., Meschia J.F., Zubair A.C. (2019). Safety and Efficacy of Intraventricular Delivery of Bone Marrow-Derived Mesenchymal Stem Cells in Hemorrhagic Stroke Model. Sci. Rep..

[168] Jayaram P., Ikpeama U., Rothenberg J.B., Malanga G.A. (2019). Bone Marrow–Derived and Adipose-Derived Mesenchymal Stem Cell Therapy in Primary Knee Osteoarthritis: A Narrative Review. PMR.

[169] Murphy M.P., Buckley C., Sugrue C., Carr E., O’reilly A., O’neill S., Carroll S.M. (2017). ASCOT: Autologous Bone Marrow Stem Cell Use for Osteoarthritis of the Thumb—First Carpometacarpal Joint. Plast. Reconstr. Surg. Glob. Open.

[170] Campbell T.M., Churchman S.M., Gomez A., McGonagle D., Conaghan P.G., Ponchel F., Jones E. (2016). Mesenchymal stem cell alterations in bone marrow lesions in patients with hip osteoarthritis. Arthritis Rheumatol..

[171] Kong L., Zheng L.-Z., Qin L., Ho K.K.W. (2017). Role of mesenchymal stem cells in osteoarthritis treatment. J. Orthop. Transl..

[172] Soler Rich R., Munar A., Soler Romagosa F., Peirau X., Huguet M., Alberca M. (2015). Treatment of knee osteoarthritis with autologous expanded bone marrow mesenchymal stem cells: 50 cases clinical and MRI results at one year follow-up. J. Stem Cell Res. Ther..

[173] Lin H., Sohn J., Shen H., Langhans M.T., Tuan R.S. (2019). Bone marrow mesenchymal stem cells: Aging and tissue engineering applications to enhance bone healing. Biomaterials.

[174] Freitag J., Bates D., Boyd R., Shah K., Barnard A., Huguenin L., Tenen A. (2016). Mesenchymal stem cell therapy in the treatment of osteoarthritis: Reparative pathways, safety and efficacy—A review. BMC Musculoskelet. Disord..

[175] Pas H.I., Winters M., Haisma H.J., Koenis M.J.J., Tol J.L., Moen M.H. (2017). Stem cell injections in knee osteoarthritis: A systematic review of the literature. Br. J. Sports Med..

[176] Iijima H., Isho T., Kuroki H., Takahashi M., Aoyama T. (2018). Effectiveness of mesenchymal stem cells for treating patients with knee osteoarthritis: A meta-analysis toward the establishment of effective regenerative rehabilitation. NPJ Regen. Med..

[177] Tucker B.A., Karamsadkar S.S., Khan W.S., Pastides P. (2010). The role of bone marrow derived mesenchymal stem cells in sports injuries. J. Stem Cells.

[178] Gobbi A., Fishman M. (2016). Platelet-rich plasma and bone marrow–derived mesenchymal stem cells in sports medicine. Sports Med. Arthrosc. Rev..

[179] Muroi K., Miyamura K., Okada M., Yamashita T., Murata M., Ishikawa T., Uike N., Hidaka M., Kobayashi R., Imamura M. (2016). Bone marrow-derived mesenchymal stem cells (JR-031) for steroid-refractory grade III or IV acute graft-versus-host disease: A phase II/III study. Int. J. Hematol..

[180] Introna M., Lucchini G., Dander E., Galimberti S., Rovelli A., Balduzzi A., Longoni D., Pavan F., Masciocchi F., Algarotti A. (2014). Treatment of Graft versus Host Disease with Mesenchymal Stromal Cells: A Phase I Study on 40 Adult and Pediatric Patients. Biol. Blood Marrow Transplant..

[181] Shenoy S., Eapen M., Panepinto J.A., Logan B.R., Wu J., Abraham A., Brochstein J., Chaudhury S., Godder K., Haight A.E. (2016). A trial of unrelated donor marrow transplantation for children with severe sickle cell disease. Blood.

[182] Carlsson P.-O., Schwarcz E., Korsgren O., Le Blanc K. (2015). Preserved β-cell function in type 1 diabetes by mesenchymal stromal cells. Diabetes.

[183] Bhansali S., Dutta P., Kumar V., Yadav M.K., Jain A., Mudaliar S., Bhansali S., Sharma R.R., Jha V., Marwaha N. (2017). Efficacy of autologous bone marrow-derived mesenchymal stem cell and mononuclear cell transplantation in type 2 diabetes mellitus: A randomized, placebo-controlled comparative study. Stem Cells Dev..

[184] Ciccocioppo R., Gallia A., Sgarella A., Kruzliak P., Gobbi P.G., Corazza G.R. (2015). Long-Term Follow-Up of Crohn Disease Fistulas After Local Injections of Bone Marrow–Derived Mesenchymal Stem Cells. Mayo Clin Proc..

[185] Molendijk I., Bonsing B.A., Roelofs H., Peeters K.C.M.J., Wasser M.N.J.M., Dijkstra G., van der Woude C.J., Duijvestein M., Veenendaal R.A., Zwaginga J.-J. (2015). Allogeneic bone marrow–derived mesenchymal stromal cells promote healing of refractory perianal fistulas in patients with Crohn’s disease. Gastroenterology.

[186] Dhere T., Copland I., Garcia M., Chiang K.Y., Chinnadurai R., Prasad M., Galipeau J., Kugathasan S. (2016). The safety of autologous and metabolically fit bone marrow mesenchymal stromal cells in medically refractory Crohn’s disease–a phase 1 trial with three doses. Aliment. Pharmacol. Ther..

[187] Steinberg G.K., Kondziolka D., Wechsler L.R., Lunsford L.D., Coburn M.L., Billigen J.B., Kim A.S., Johnson J.N., Bates D., King B. (2016). Clinical outcomes of transplanted modified bone marrow–derived mesenchymal stem cells in stroke: A phase 1/2a study. Stroke.

[188] Hare J.M., DiFede D.L., Rieger A.C., Florea V., Landin A.M., El-Khorazaty J., Khan A., Mushtaq M., Lowery M.H., Byrnes J.J. (2017). Randomized comparison of allogeneic versus autologous mesenchymal stem cells for nonischemic dilated cardiomyopathy: POSEIDON-DCM trial. J. Am. Coll. Cardiol..

[189] Cai M., Shen R., Song L., Lu M., Wang J., Zhao S., Tang Y., Meng X., Li Z., He Z.-X. (2016). Bone marrow mesenchymal stem cells (BM-MSCs) improve heart function in swine myocardial infarction model through paracrine effects. Sci. Rep..

[190] Wilson J.G., Liu K.D., Zhuo H., Caballero L., McMillan M., Fang X., Cosgrove K., Vojnik R., Calfee C.S., Lee J.-W. (2015). Mesenchymal stem (stromal) cells for treatment of ARDS: A phase 1 clinical trial. Lancet Respir. Med..

[191] Suk K.T., Yoon J.H., Kim M.Y., Kim C.W., Kim J.K., Park H., Hwang S.G., Kim D.J., Lee B.S., Lee S.H. (2016). Transplantation with autologous bone marrow-derived mesenchymal stem cells for alcoholic cirrhosis: Phase 2 trial. Hepatology.

[192] Song Y.M., Lian C.H., Wu C.S., Ji A.F., Xiang J.J., Wang X.Y. (2015). Effects of bone marrow-derived mesenchymal stem cells transplanted via the portal vein or tail vein on liver injury in rats with liver cirrhosis. Exp. Ther. Med..

[193] Lin B.L., Chen J.F., Qiu W.H., Wang K.W., Xie D.Y., Chen X.Y., Liu Q.L., Peng L., Li J.G., Mei Y.Y. (2017). Allogeneic bone marrow–derived mesenchymal stromal cells for hepatitis B virus–related acute-on-chronic liver failure: A randomized controlled trial. Hepatology.

[194] Luo D., Hu S.Y., Liu G.X. (2018). Multi-channel promotion of lung cancer progress by bone marrow derived mesenchymal stem cells in tumor microenvironment. Chin. J. Oncol..

[195] Zhang X., Sai B., Wang F., Wang L., Wang Y., Zheng L., Li G., Tang J., Xiang J. (2019). Hypoxic BMSC-derived exosomal miRNAs promote metastasis of lung cancer cells via STAT3-induced EMT. Mol. Cancer.

[196] Papaccio F., Paino F., Regad T., Papaccio G., Desiderio V., Tirino V. (2017). Concise review: Cancer cells, cancer stem cells, and mesenchymal stem cells: Influence in cancer development. Stem Cells Transl. Med..

[197] Zhao Y., Lai W., Xu Y., Li L., Chen Z., Wu W. (2013). Exogenous and endogenous therapeutic effects of combination Sodium Ferulate and bone marrow stromal cells (BMSCs) treatment enhance neurogenesis after rat focal cerebral ischemia. Metab. Brain Dis..

[198] Tsai L.-K., Wang Z., Munasinghe J., Leng Y., Leeds P., Chuang D.-M. (2011). Mesenchymal Stem Cells Primed With Valproate and Lithium Robustly Migrate to Infarcted Regions and Facilitate Recovery in a Stroke Model. Stroke.

[199] Esneault E., Pacary E., Eddi D., Freret T., Tixier E., Toutain J., Touzani O., Schumann-Bard P., Petit E., Roussel S. (2008). Combined Therapeutic Strategy Using Erythropoietin and Mesenchymal Stem Cells Potentiates Neurogenesis after Transient Focal Cerebral Ischemia in Rats. J. Cereb. Blood Flow Metab..

